# Retinal Electrophysiological Effects of Intravitreal Bone Marrow Derived Mesenchymal Stem Cells in Streptozotocin Induced Diabetic Rats

**DOI:** 10.1371/journal.pone.0156495

**Published:** 2016-06-14

**Authors:** Eren Çerman, Tolga Akkoç, Muhsin Eraslan, Özlem Şahin, Selvinaz Özkara, Fugen Vardar Aker, Cansu Subaşı, Erdal Karaöz, Tunç Akkoç

**Affiliations:** 1 Marmara University School of Medicine, Department of Ophthalmology, Istanbul, Turkey; 2 Genetic Engineering and Biotechnology Institution, The Scientific and Technological Research Council of Turkey, Kocaeli, Turkey; 3 Haydarpaşa Numune Education and Research Hospital, Department of Pathology, Istanbul, Turkey; 4 Kocaeli University Center for Stem Cell and Gene Therapies, Kocaeli, Turkey; 5 Marmara University School of Medicine, Department of Pediatric Allergy and Immunology, Istanbul, Turkey; Queen's University Belfast, UNITED KINGDOM

## Abstract

Diabetic retinopathy is the most common cause of legal blindness in developed countries at middle age adults. In this study diabetes was induced by streptozotocin (STZ) in male Wistar albino rats. After 3 months of diabetes, rights eye were injected intravitreally with green fluorescein protein (GFP) labelled bone marrow derived stem cells (BMSC) and left eyes with balanced salt solution (Sham). Animals were grouped as *Baseline* (n = 51), *Diabetic* (n = 45), *Diabetic+BMSC* (n = 45 eyes), *Diabetic+Sham* (n = 45 eyes), *Healthy+BMSC* (n = 6 eyes), *Healthy+Sham* (n = 6 eyes). Immunohistology analysis showed an increased retinal gliosis in the *Diabetic* group, compared to *Baseline* group, which was assessed with GFAP and vimentin expression. In the immunofluorescence analysis BMSC were observed to integrate mostly into the inner retina and expressing GFP. *Diabetic* group had prominently lower oscillatory potential wave amplitudes than the Baseline group. Three weeks after intravitreal injection *Diabetic+BMSC* group had significantly better amplitudes than the *Diabetic+Sham* group. Taken together intravitreal BMSC were thought to improve visual function.

## Introduction

Diabetes, age-related macular degeneration and glaucoma are the most common causes of legal blindness in developed countries.[1] The common pathways in these conditions consist of the progressive loss of photoreceptors, interneurons, glial cells and ganglion cells.

Despite of the prominent progress in ophthalmology, the World Health Organization estimated that diabetic retinopathy (DR) is responsible for 4.8% of the 37 million cases of blindness throughout the world. Although some animals like amphibians have the capacity to regenerate complete retina throughout their lives, [2, 3] mature mammalian eyes are thought to lack any retinal regenerative capacity. Stem cell treatments, while promising, are still at early experimental stages in ophthalmology.

Stem cells have the capacity to generate different types of daughter cells with asymmetric mitotic division, and thus they are accepted as an easy tool for regeneration of damaged tissue. Many types of stem cells such as embryonic stem cells [4, 5] hematopoietic stem cells, [6] endothelial progenitor cells [7] induced pluripotent stem cells [5, 8, 9] umbilical cord blood derived myeloid progenitor cells, [10] and mesenchymal stem cells [11, 12] are implicated in various types of retinopathies. [13, 14]

Mesenchymal stem cells (MSC) are ubiquitously found in almost all tissues in the body and migrate into the nervous system in response to injury. They can differentiate into fully functional neurons, [15] but their benefits may also arise from the production of neurotrophic factors and the repair of the vasculature, which is equally observed in MSC isolated from various tissues. [16] They can be isolated from cord blood, Wharton’s jelly, the placenta, bone marrow, teeth, and adipose tissue, which makes them favorable for autologous transplantation. As a promising therapeutic tool to suppress inflammation and immunomodulation, bone marrow derived mesenchymal stem cells (BMSC) have also been widely used in preclinical treatment studies of several autoimmune disorders.[15–21].

Among these cells, intravitreal injection of adipose derived MSC have been demonstrated to be possibly effective in pericyte replacement, [12] improving blood retina barrier integrity and differentiating into photoreceptor cells or astrocytes in streptozotocin (STZ) induced diabetic retinopathy models. [17]

An improvement in functional vision has been shown with retinal progenitor cells which migrate into retina and differentiate to mature retinal cells.[18] The fundamental question whether stem cells that integrate into the retina can create a functional vision in totally blind subjects by forming new synapses, was answered in a study, where functional vision was evidenced after rod precursor transplantation in adult Gnat1^−/−^ mice, totally lacking rod function. [19]

On the other hand bone marrow-derived mesenchymal stem cells (BMSC) are relatively easily isolated than the retinal progenitor cells or induced pluripotent stem cells. They have been shown to inhibit photoreceptor apoptosis and slow down retinal damage *in vivo* and *in vitro* by expressing bFGF and BDNF. [20] Intraocular transplantation of BMSC can prevent retinal ganglion cell apoptosis in optic nerve injury or glaucoma models, [21, 22] and are shown to differentiate into photoreceptors in vivo and in vitro. [23]

To assess their possible functional effect in restoring vision, in this study, we evaluated the change in electroretinography (ERG) after intravitreal injection of rat BMSC in a streptozotocin (STZ) induced diabetes model; examined the migration of green fluorescein protein (GFP) labeled BMSC into the retina by immunofluorescence, assessed the degree of reactive gliosis in STZ induced diabetic retinopathy by immunohistochemistry with vimentin and glial fibrillary acidic protein (GFAP) antibodies, which was shown to be increased in diabetic retinopathy in previous studies, [24–26] and assessed any change in gliosis after intravitreal BMSC injection.

## Materials and Methods

### Animals and experimental design

Ethics approval was granted by the Committee of Ethics in Animal Experimentation of Marmara University, Istanbul, Turkey and is in compliance with the ARVO Statement for the Use of Animals in Ophthalmic and Vision Research

A total of 8 weeks old 60 male albino rats of Wistar strain weighing about 200–250 g were enrolled into the study. These rats were born and reared in Turkish Genetic Engineering and Biotechnology Institute Laboratory Animal Breeding Facility (TUBITAK MAM), where the temperature was maintained at 21°C with 12-h light/ dark cycles, 60% humidity atmosphere, and animals had unrestricted access to rat pellet diet and water. Rats’ cases exchanged minimum every other day. At the end of the study rats were sacrificed humanely by overdose anesthetics. None of the rats had clinical signs of any suffering. During the course of the study 8 rats died due to anesthetics, and euthanasia was performed for one rat that developed cataracts. In this study the data of remaining 51 animals are presented.

**Fig 1:**
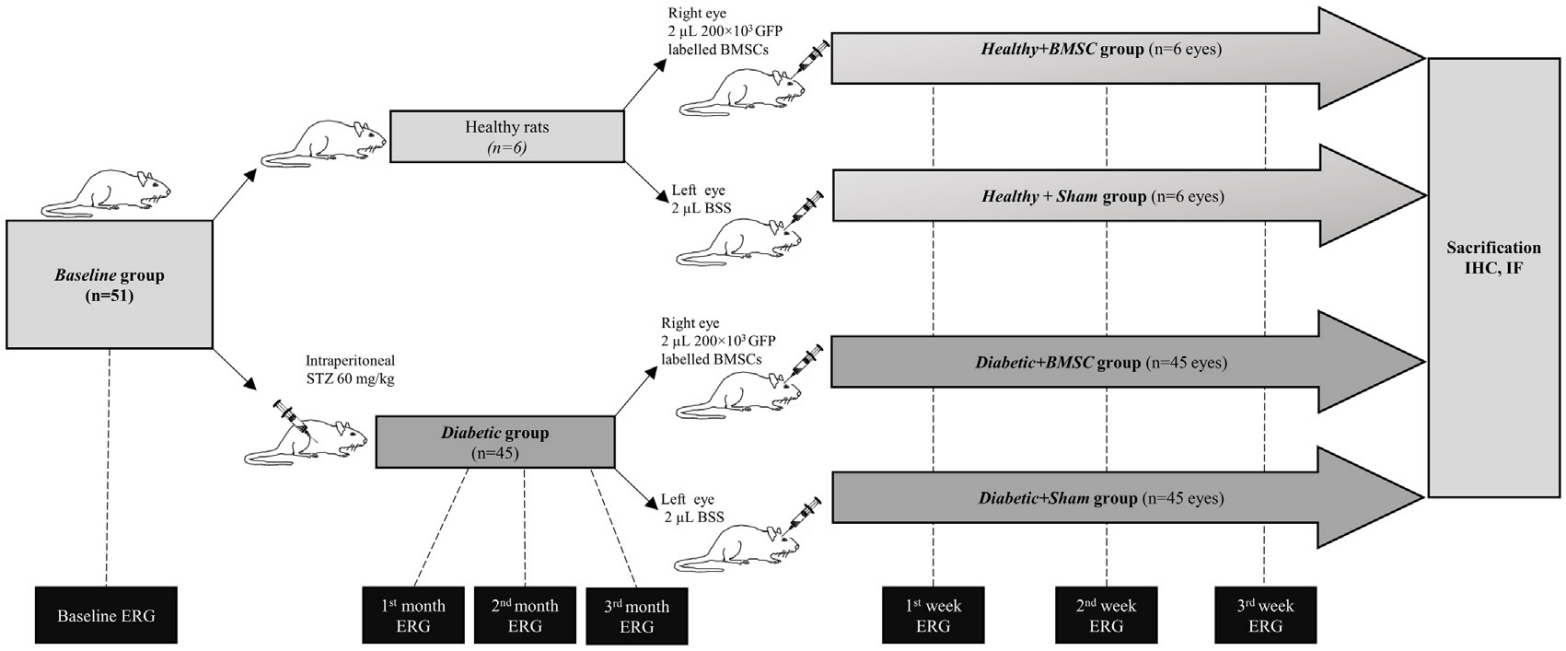
Main study design. All rats underwent a baseline ERG which were labelled as *Baseline* group. Among these animals 46 underwent intraperitoneal STZ injection and were labelled as *Diabetic* group afterwards for induced diabetes, the rest did not receive STZ. Diabetic animals were followed for 3 months. At the end of the three months all rats received intravitreal 20x103 bone marrow derived stem cell containing solution into the right eyes and balanced salt solution into the left eyes to make up the *Healthy +BMSC*, *Healthy + Sham*, *Diabetic +BMSC* and *Healthy + Sham* groups. These groups underwent ERG analysis at 1^st^ week, 1^st^ week and 2^nd^ week after the intravitreal injection and were sacrificed thereafter.

**Fig 2:**
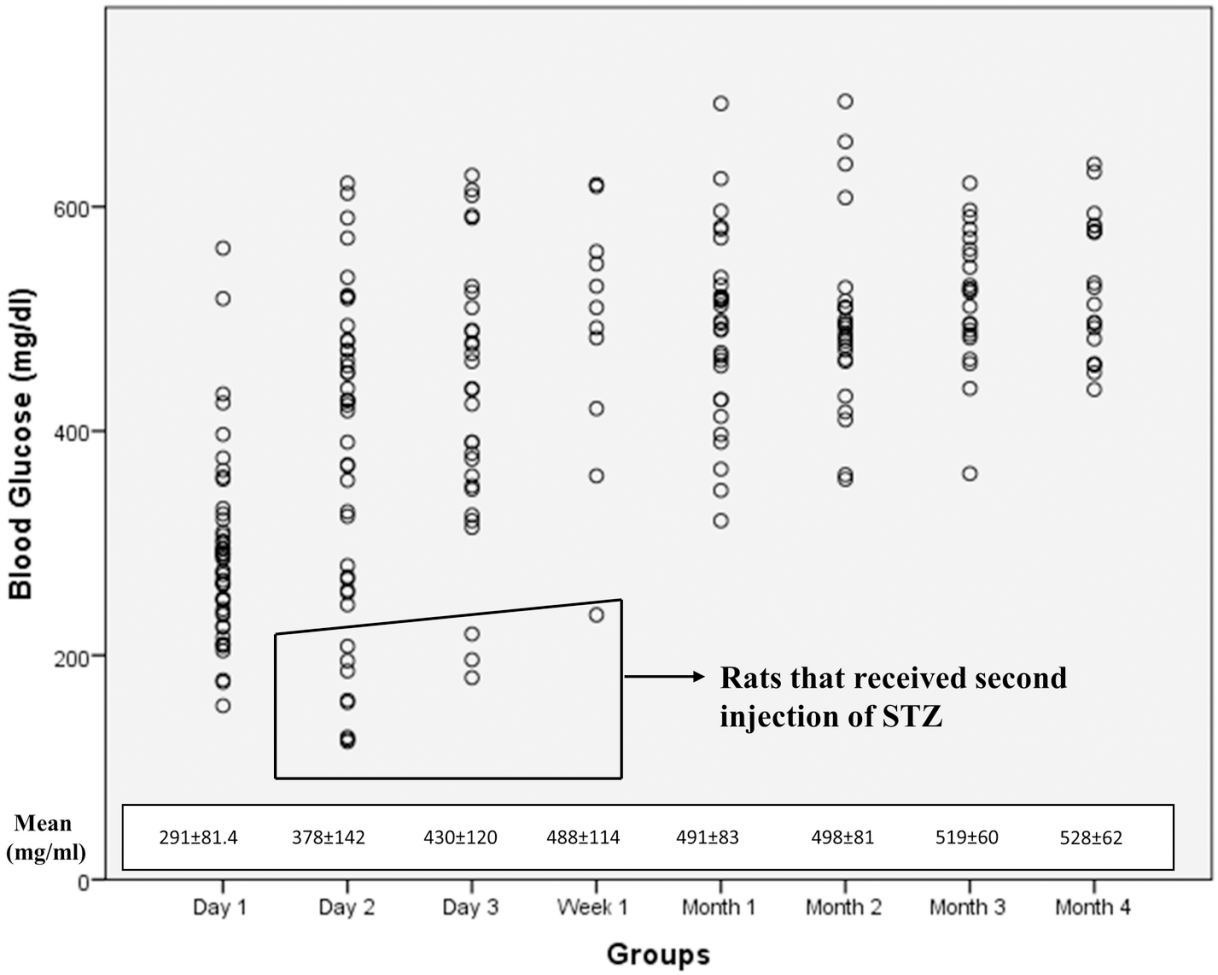
The mean blood glucose levels of *Diabetic* group, *Diabetic+BMSC* and *Diabetic+Sham* groups. Note that the right eyes of the *Diabetic* group were injected with BMSC to create *Diabetic+BMSC* group, and sham to create *Diabetic+Sham* group. The blood glucose levels after the treatment with BMSC or Sham are shown as 4^th^ month.

The study design is illustrated on Fig 1. The baseline ERG of 51 animals were obtained from both eyes and this group was labelled as the "*Baseline"* group. Among these animals 45 received an intraperitoneal injection of 60 mg/kg of streptozotocin (STZ) (Sigma Aldrich Co., St. Louis, MO, USA) to induce diabetes, as was described in previous studies. [17, 27, 28] The blood glucose levels measured during the course of the study are given in Fig 2. 12 rats received a second dose. The rats having a blood glucose level higher than 200 mg/ml in two different measurements in the first three days of STZ injections were accepted as *Diabetic* group and ERG measurements from both eyes were obtained at 1^st^, 2^nd^ and 3^rd^ month of induced diabetes.

At the end of the 3^rd^ month all animals in the *Diabetic* group received a 2 μL solution containing 200×10^3^ GFP labelled BMSC into the rats’ right eye with a 30-gauge Hamilton syringe. These eyes made up the "*Diabetic+BMSC*" group. Simultaneously the left eyes were injected with an equal volume of balanced salt solution (Miray Medikal, Bursa, Turkey) as sham to recruit the "*Diabetic+Sham"* group.

Six animals from the baseline group did not receive a STZ injection and were accepted as healthy. They similarly received a 2 μL solution containing 200×10^3^ GFP labelled BMSC into the right eyes in order to establish the *Healthy+BMSC* group and an equal volume of balanced salt solution into the left eye to create the *Healthy+Sham* group. The intravitreal injections were performed under binocular stereomicroscope (Tronic XTX 3C, Beijing, People’s Republic of China) through the cornea-scleral limbus with the bevel up. When the needle reached the vitreous, the material was slowly and progressively injected while avoiding any contact with the lens.

Post-injection ERG analyses were performed at 1^st^ week, 2^nd^ week and 3^rd^ week of intravitreal injection in *Diabetic+BMSC*, *Diabetic+Sham*, *Healthy+BMSC* and *Healthy+Sham* groups. At the end of the study all rats were sacrificed, both eyes were collected and GFAP, Rhodopsin, Vimentin and BRN3a and localization of GFP positive BMSC were analyzed with immunoflourescence and immunohistochemistry.

### Isolation and preparation of GFP labelled rat BMSC

#### Isolation

In order to obtain rat BMSC, healthy Wistar albino rats apart from the study subjects were sacrificed with an overdose of hydrochloride and xylazine, and bone marrow from the femur and tibias were extracted.

Dissected femurs and tibias were put in 70% isopropanol for a few seconds, transferred to 1X D-PBS, then to a 10 cm dish containing Dulbecco’s modified Eagle Medium (DMEM). Each bone was then held with forceps and the two ends were cut to open with a scissor. The syringe was filled with DMEM and the marrow was flushed into a 50 ml tube by inserting a needle to the open end of the bone. This process was repeated for 3 times for each bone. Thereafter cells were resuspended with DMEM and passed through a 70 μm cell strainer to remove the bone debris and blood aggregates. Cells were centrifuged at 200g, 4°C for 5 minutes and the supernatant was discarded. Cells were resuspended in 25 ml MSC medium (DMEM 10% FBS and 1% penicillin/streptomycin). 10ml cell suspensions were cultivated in T-25 flasks in a 5% CO2 atmosphere under 37°C incubator. The stem cells were washed with DPBS and provided with fresh culture medium. The culture medium was changed every 3 to 4 days until the cells reached confluence. The cells were detached with 0.25% trypsin EDTA (Gibco, USA) when reached 70–80% confluence. Adherent cells were cultured for 3 passages and were analyzed for specific surface markers.

#### Characterization

The cellular differentiation analysis was performed using flow cytometry. To analyze the cell surface antigen expressions, the cells after third passage was used. MSCs were incubated with antibodies for rat CD90 FITC (BD Biosciences, San Diego, CA, USA), CD 29 FITC (BD Biosciences, San Diego, CA, USA), CD106 PE (BD Biosciences, San Diego, CA, USA), CD54 PE (BD Biosciences, San Diego, CA, USA) at room temperature in the dark. Control antibodies were FITC Rat IgG2a, K isotype controls and IgG1 PE isotype controls (BD Biosciences, San Diego, CA, USA). Negative markers were CD3 PE (BD Biosciences, San Diego, CA, USA), CD4 APC (BD Biosciences, San Diego, CA, USA), CD25 FITC (BD Biosciences, San Diego, CA, USA), CD45 FITC (BD Biosciences, San Diego, CA, USA), CD8B FITC (BD Biosciences, San Diego, CA, USA).

#### Differentiation

Osteogenic differentiation (StemPro^®^ Osteogenesis Differentiation Kit Gibco), adipogenic differentiation (StemPro^®^ Adipogenesis Differentiation Kit Gibco) and chondrogenic differentiation (StemPro^®^ Chondrogenesis Differentiation Kit Gibco) were carried out. Rat BMSC functional identification kit (Gibco, Grand Island, USA) was used. For differentiation process, the cells were plated in 6-well plates (5 × 10^4^ cell/well), and the differentiation medium was prepared according to the manufacturer’s instructions and changed three times per week. After 14 days, the adipocytes and chondrocytes were stained with Oil Red O and Alcian blue, respectively, and after 28 days, the osteocytes were stained with Alizarin red.

#### GFP vector transfection

**Fig 3:**
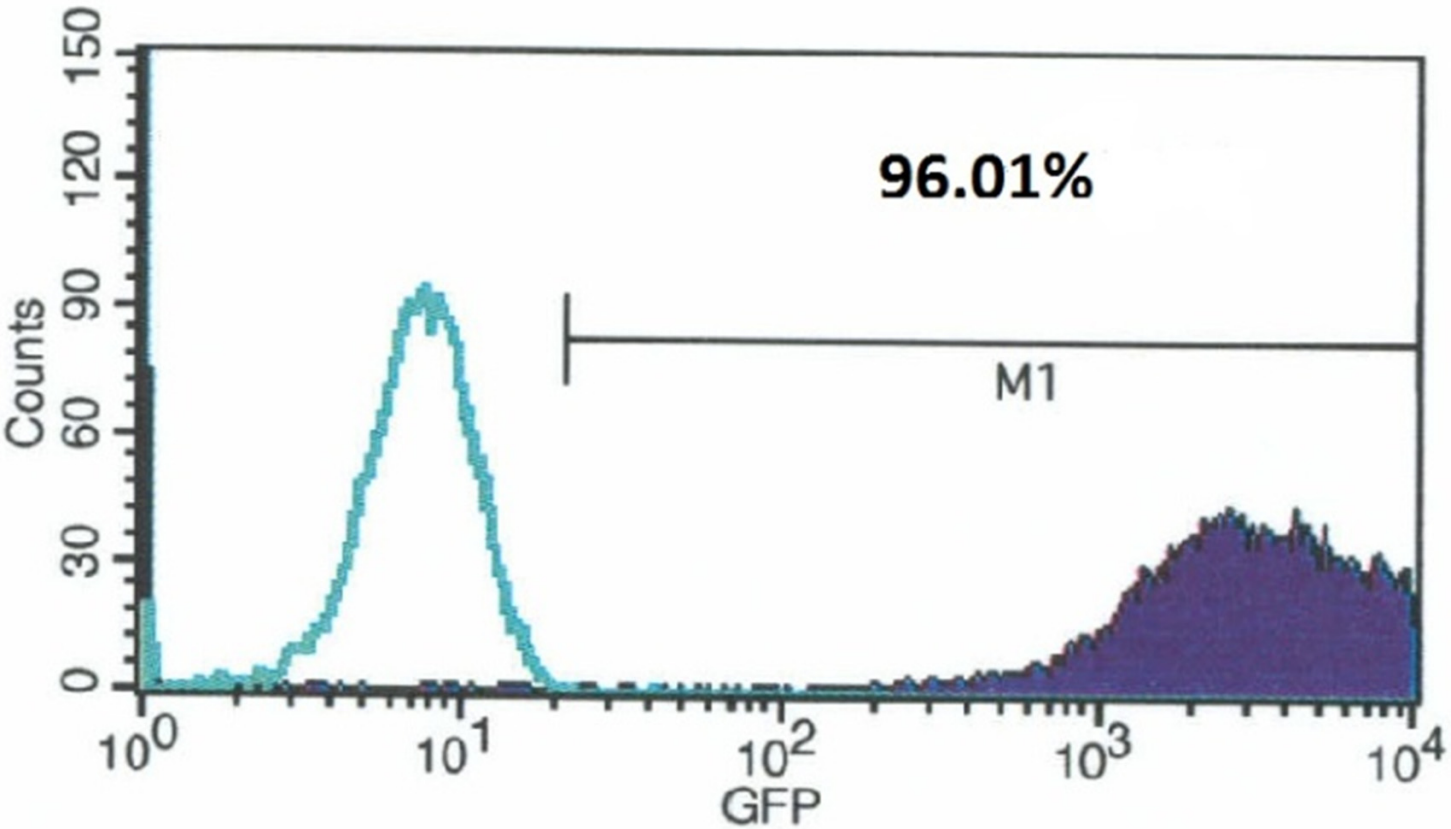
Within the histogram of GFP-transfected cells, the green line shows non-transfected cells and the region(M1) to the right of the vertical line represents the GFP fluorescence. The transfection efficiency and mean expression level can then be calculated by the CellQuest Pro Software.

According to the manufacturers instructions BMSC on third passage were transfected with GFP vector (pJTI^™^ R4 Dest CMV N-EmGFP pA Vector, Thermofisher) to express green fluorescent protein. One day before transfection, the cells in 35 mm culture dish were plated in 2 ml growth medium without antibiotics and that cells were 90–95% confluent at the time of transfection. 4.0 μg GFP vector was diluted in 250 μl of RPMI 1640 medium without serum. The cells were incubated at 37°C in a 5% CO_2_ incubator for 24–48 hours until they were ready to assay for transgene GFP expression. The flow cytometry results showing the percentage of GFP transfected cells are given in Fig 3.

#### Obtaining and reliability of GFP labelled stem cells

**Fig 4:**
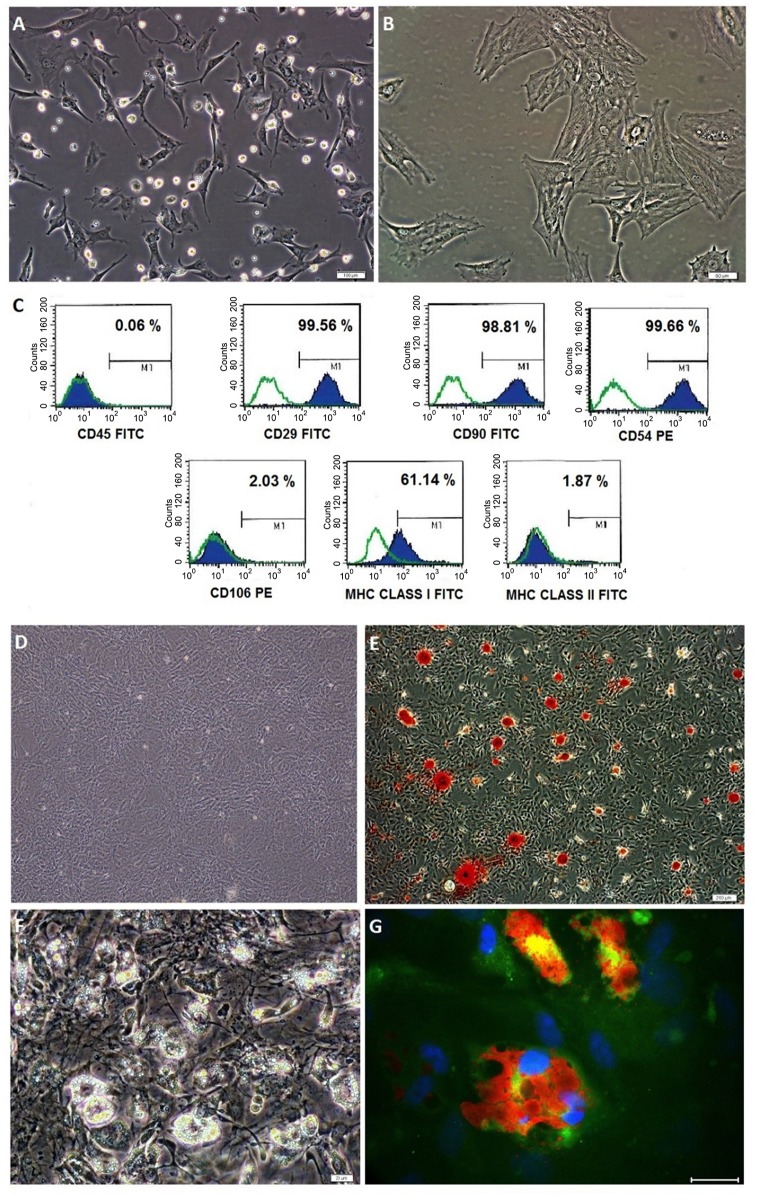
Bone marrow derived mesenchymal stem cell (BMSC) morphologies for primary culture (A) Passage 0, (B) Passage 3; Flow cytometry analysis for BMSC (C); Differentiation of BMSC into osteogenic, staining with Alizarin Red-S for control (D) and differentiation group (E) and adipogenic differentiation of BMSC (F,G) staining with Red-Oil (G). (Scale bar, 50 μm).

During the isolation, most of the attached cells on the culture flasks displayed fibroblast-like, spindle-shaped formation during the early days of incubation. These cells began to proliferate after 3–4 days of incubation and gradually grew to form small colonies (Fig 4A). After plating for 11–15 days, these primary cells reached 70–80% confluence. At third passage, the majority of these stem cells exhibited large, flattened or fibroblast-like morphology (Fig 4B). Flow cytometric analyses of the BMSC revealed the existence of previously defined markers of BMSC. The data indicated that the rat BMSC expressed CD29, CD54, CD90 and MHC Class I but not MHC Class II, CD45 and CD106 (Fig 4C). The findings were consistent with the undifferentiated state of the cells possessing immunophenotypic characteristics of BMSC. Cells were stored at -80°C for a longer period demonstrated a high vitality and a capability to quickly restart proliferation. These cells were capable of differentiating into adipocytes and osteoblasts (Fig 4D–4G).

### Electroretinography

For all ERG measurement session dark adaptation was obtained overnight (12 hours) and all process was performed under a dim red light. The pupils were dilated with 1.5% cyclopentolate (Abdi İbrahim, Turkey) and 0.5% tropicamide (Bilim İlaç, Turkey). Before the session rats were anesthetized with intraperitoneal ketamine hydrochloride (100 mg/kg), xylazine hydrochloride (5 mg/kg) combination and the eyes were lubricated with a carbomer liquid gel (Viscotears^®^). Rats were placed on a custom made wooden plate with a tissue and silver wire-loop DTL electrodes were fit into each conjunctival sac at 360°. Two needle reference electrodes were placed subcutaneously behind each ear and the ground electrode was placed in the tail. ERG was recorded and analyzed with the computerized Opto-electronic Stimulator Vision Monitor Mon-Pack 120 Metrovision (Pérenchies, France). The responses were amplified by 5,000x, and they were high-pass filtered with a 1 Hz cutoff frequency and low-pass filtered at 300 Hz. The light source was a 100W tungsten–halogen lamp, which was placed 1 cm from the face of the rat. The stimulus duration was 2 ms. The interstimulus duration was 8 seconds. Four increasing stimulus intensities of 0.0122 cd.s/m^2^, 0.244 cd.s/m^2^, 0.975 cd.s/m^2^, and 3.41 cd.s/m^2^ were used. The mean amplitudes of eight consecutive flashes were recorded in each measurement.

#### Plotting

Plots of the mean ERG waveforms (a, b, and OP) in different groups were created with the help of Microsoft Excel (Redmond, Washington: Microsoft, 2013) using the previously described formula for ERG waves. During the plotting of intensity response functions sum-of-squares merit function using Solver function in Microsoft Excel was used.

The ERG waves were plotted as the sum of PIII or “a” wave, PII or “b” wave and an OP wave. The “a” wave was plotted with the delayed Gaussian formula, as was described previously. [29] [30] The “b” wave is described previously as the sum of a logistic growth function and a Gaussian function, which creates delayed function. [31] Similarly we used a delayed Gaussian function also for the “b” wave.

$$Gaussian(t)\;=\;m∙e^{\left[-\;\frac{{\left(t-u\right)}^{2}}{2s^{2}}\right]}$$

Here m defines the maximum amplitude (microvolts), u the peak time (ms), and **s** the spread of the Gaussian function. The beginning of the function was accepted as null as a rule for Solver. The spread of the Gaussian function was calculated as s = u, where the m was defined with an x constant.

Oscillatory potential waves were measured beginning from the negative peak to the next positive peak, where all peaks were marked as OP1, OP2 OP3 and OP4 respectively and were added up as SumOP automatically by the device, as previously recommended [32][33].OP waves were modeled with the Gabor function, as was described previously: [34, 35][36]

$$Gabor(t)\;=\;a∙e^{-\pi [{\left(\frac{t-m}{s}\right)}^{2}]}∙cos(2\pi f(t-m)+\phi ).$$

The intensity response function was described with the Naka–Rushton equation, as was described previously: [37][38]

R = Rmax Ln/(Ln+Kn)

where *R* is the response to a stimulus of luminance L, *R*_*max*_ is the maximum response amplitude, K is the luminance required to elicit the half-amplitude of *R*_*max*._,and n is a constant proportional to the slope of the graph at point K. When the equation was fitted to the data, *R*_*max*_, *K*,and *n* were estimated for each individual graph with Solver function.

### Pathologic analyses

The rats were deeply anesthetized with ketamine hydrochloride (100 mg/kg) and xylasine (5 mg/kg), and they were intra-cardially perfused with 4% paraformaldehyde. Their eyes were enucleated and immersion fixed overnight in 4% paraformaldehyde in PBS (pH 7.4) at 4°C. They were subsequently dehydrated over several hours and embedded in paraffin in transverse orientation.

#### Immunohistochemistry

The aim of immunohistochemistry was to assess the level gliosis in *Diabetic+Sham*, *Diabetic+BMSC* and compare with their *Healthy+Sham* and *Healthy+BMSC* counterparts. These samples were processed and scored for retinal gliosis in a blind manner.

The enucleation material was fixated in formalin, embedded in paraffin blocks, and cut in 4 μm parallel layers that passed through the optic disc and pupil. The specimens were stained with hematoxylin and eosin for histopathologic examination. For the immunohistochemical examination, the samples were deparaffinized. All primary antibodies, secondary antibodies and dilution material were obtained from Abcam, (Cambridge, MA, USA). Anti-rhodopsin antibody, ab98887; anti-GFAP antibody, ab4674; anti-vimentin antibody; ab8979; secondary antibodies; ab150120, ab150170, and ab150120 and dilution material, ab64211. The samples were incubated with streptavidin-conjugated peroxidase (ab64269) for 45 minutes and stained with AEC substrate. The samples were later counter-stained with Mayer’s hematoxylin.

Vimentin and GFAP expression was scored manually between 0–3.

#### Immunofluorescence

To detect GFP-labeled BMSC, paraffin-embedded sections were twice deparaffinized with xylene for 5 minutes, and rehydrated in a series of graded alcohol solutions (70%–100%). Endogenous peroxidases were inhibited by incubation with 3% H2O2 in PBS buffer. For antigen retrieval, the samples were heated to 98°C–99°C in antigen-retrieval buffer (10 mMTri-sodium citrate, 0.05% Tween 20, pH 6.0) and incubated for 30 minutes in the pressurized vessel. Nonspecific staining was blocked with a mixture of sera in 1.5%PBS for 30 minutes at room temperature, and incubated in the mixture of two primary antibodies in a pair-wise fashion with the mouse monoclonal anti-GFP antibody (SC-9996) at 1:50 dilutions for 1 hour at room temperature. Following incubation with the appropriate fluorescent-conjugated secondary antibodies, the sections were covered with mounting medium containing DAPI (Santa Cruz, Heidelberg, Germany). The cells were investigated under fluorescence microscope (Leica DMI 4000B; Leica Mycrosystems, Wetzlar, Germany). For other immunostainings, the following antibodies were supplied from Abcam (Cambridge, MA, USA): GFAP (ab4674), anti-vimentin antibody (ab8979) BRN3A (ab81213), and rhodopsin (ab3267). The dilution rate of all primary antibodies was 1:100.

### Statistics

Data were expressed as the mean ± standard error of the mean, and they were analyzed using SPSS (Statistical Package for the Social Sciences) software version 17.0 (IBM Corporation, Armonk, NY, USA). The standard *t*-test was used with a significance level of *P*<0.05. Shapiro–Wilk’s test (*P*>0.05) was performed, and a visual inspection of the histograms, Q-Q plots, box plots, skew, and kurtosis were undertaken for both the preoperative and postoperative data to differentiate between normal and non-normal distributions. As the distributions of all data were non-normal, between-group comparisons were made with the Mann–Whitney *U* test. Kruskal Wallis test was used to compare multiple groups. Wilcoxon test was applied for paired analysis.

## Results

### Immunohistochemistry

**Fig 5:**
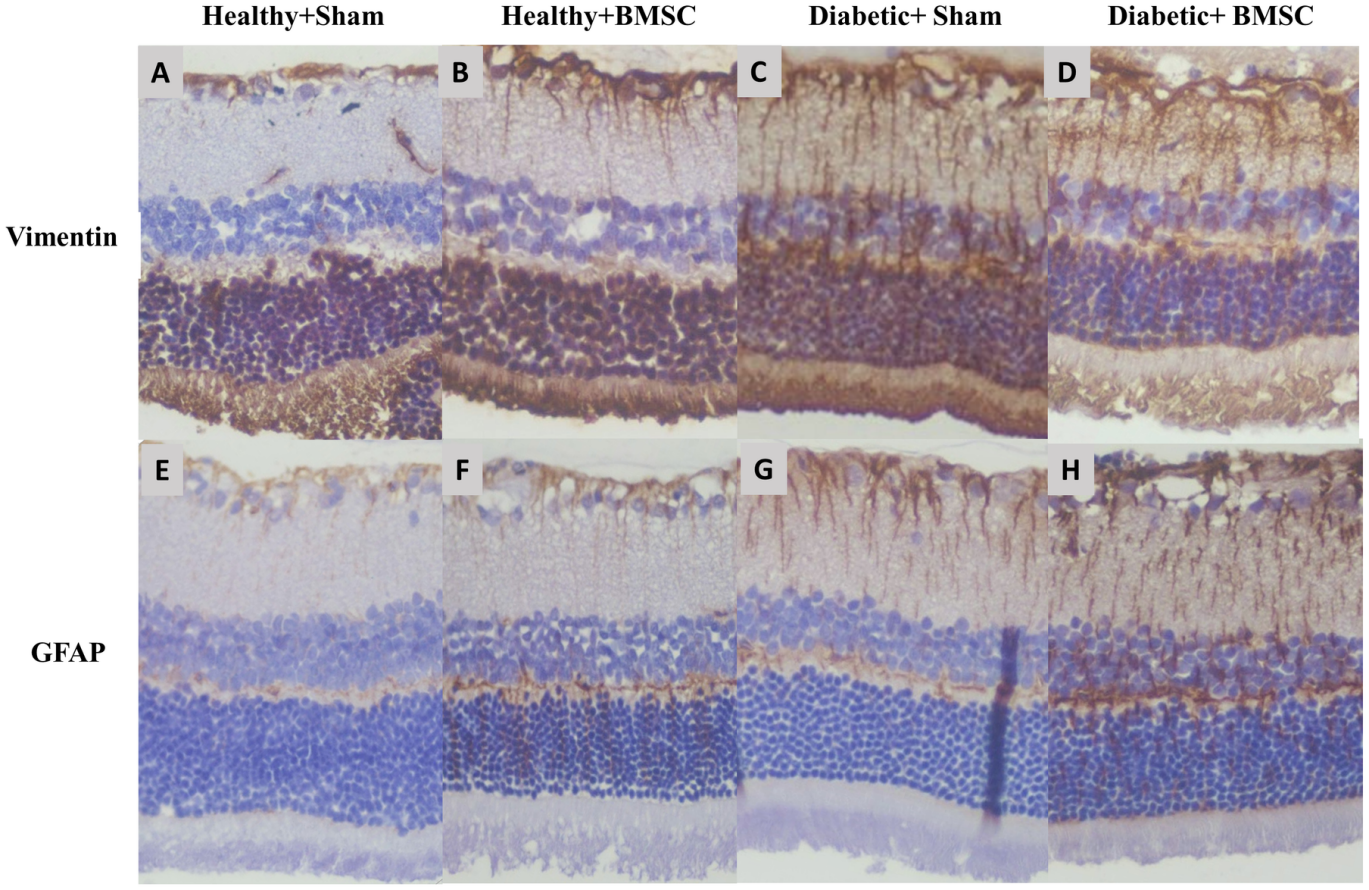
Vimentin expression in (A) *Healthy+Sham*, (B) *Healthy+BMSC*, (C) *Diabetic+Sham*, (D) *Diabetic+ BMSC* groups and GFAP expression in (E) *Healthy+Sham*, (F) *Healthy+BMSC*, (G) *Diabetic+Sham*, (H) *Diabetic+ BMSC* groups All slides were scored manually in a blind manner. The results showed an increased vimentin and GFAP expression in the diabetic groups possibly indicating an increased gliosis in diabetic animals, and a relatively reduced gliosis in *Diabetic+BMSC* groups.

A total of 22 retinas were assessed for mean vimentin and GFAP expressions with immunohistochemistry analysis. Examples of *Diabetic+Sham*, *Diabetic+BMSC*, *Healthy+Sham* and *Healthy+BMSC* groups are given in the Fig 5. The *Healthy+Sham* group had a mean vimentin expression score of 1.33 ± 0.58 (Fig 5A) and the *Healthy+BMSC* group 1.67± 0.58 (Fig 5B) where the difference was insignificant (*P*>0.05). The immunohistochemical analysis of the retinal specimens revealed that the mean vimentin expression score was 2.88± 0.35 in the *Diabetic+Sham* group (Fig 5C) and 2.13± 0.64 in the *Diabetic+BMSC* group (Fig 5D), where the difference was statistically significant (*P* < .028).

The evaluation of GFAP expression in the healthy groups revealed that the *Healthy+Sham* group had a mean expression score of 1.33±0.58 (Fig 5E) and the *Healthy+BMSC* group 1.00±0 where the difference was insignificant (Fig 5F) (*P*>0.05). The mean GFAP expression score was 2.50±0.53 in the *Diabetic+Sham* group (Fig 5G), wheras it was 2.38±0.52 in the *Diabetic+BMSC group* (Fig 5H) and the difference was insignificant (*P*>0.05).

### Immunofluorescence

**Fig 6:**
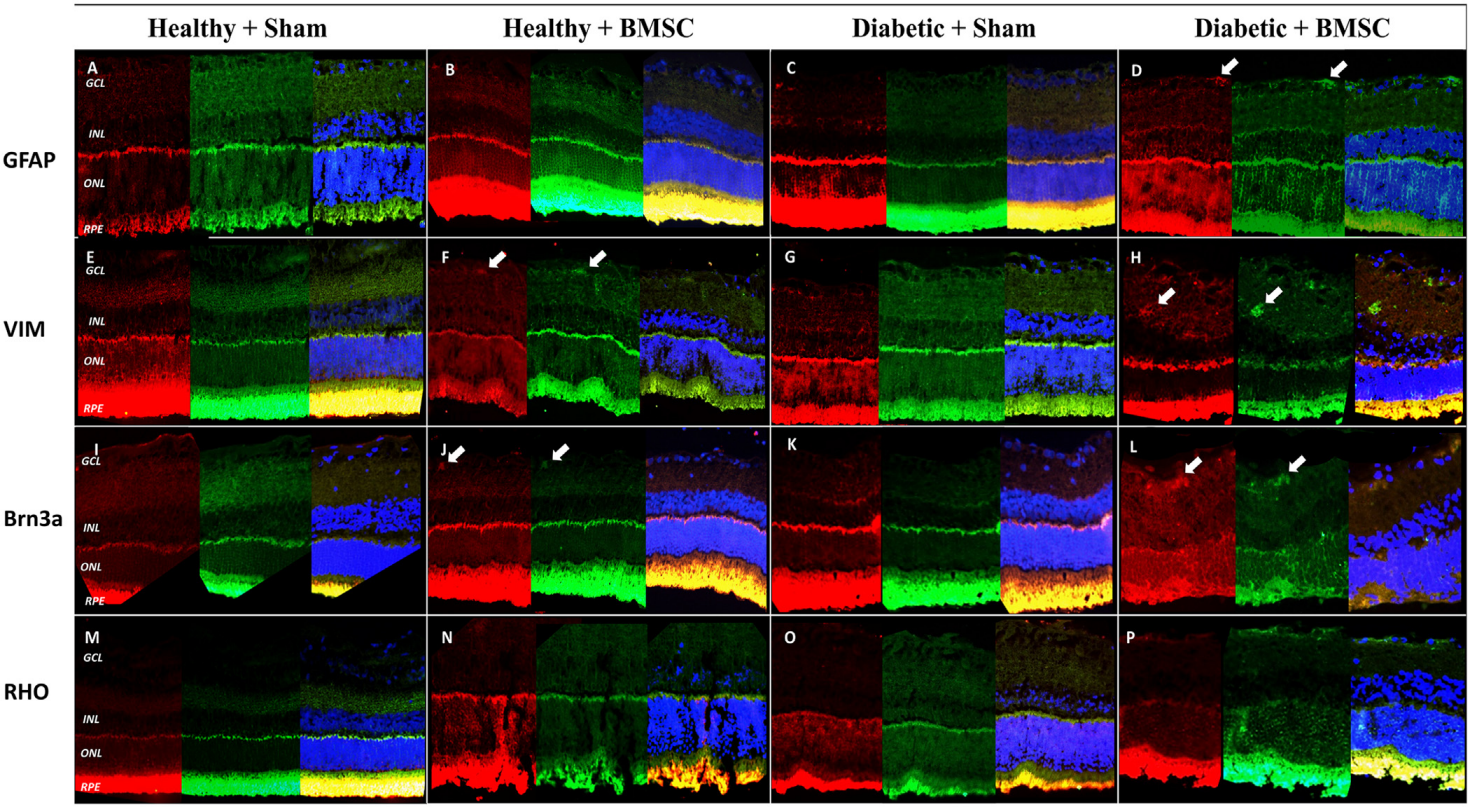
(A-D) GFAP (red), (E-H) Vimentin (red), (I-L) BRN3A (red) and (N-R) Rhodopsin (red) on GFP labelled BMSC (green) with DAPI counterstaining (blue) in the *Healthy+Sham Healthy+BMSC*, *Diabetic+Sham* and *Diabetic+BMSC* groups. In the *Diabetic+BMSC* group GFP expressing BMSC are observable throughout the retina, mostly in the inner retina. As shown in the pictures with arrow, GFP positive cells are coexpressing with vimentin, GFAP and partly BRN3A. GFAP, Glial Fibrillary Acidic Protein; VIM, vimentin; RHO, rhodopsin.

Retinal sections were analyzed for homing and differentiation potency of transplanted GFP positive BMSC. GFP positive BMSC were found mostly around inner nuclear layer (INL), ganglion cell layer (GCL) and scarcely at the outer nuclear layer (ONL) in the *Diabetic*+BMSC group. (Fig 6D, 6H, 6L and 6P)

In the *Healthy+BMSC* group some GFP labeled stem cells were also observable in the retina (Fig 6F and 6J), but it was less than in the *Diabetic+BMSC* group (Fig 6D, 6H, 6L and 6P). No GFP labelled BMSC were observable in the *Healthy+Sham* and *Diabetic+Sham* groups (Figs 6A, 6E, 6I, 6M, 6C, 6G, 6K, 6O and S1–S14)

Coexpression of GFAP with GFP (Fig 6D) and vimentin with GFP (Fig 6H) was frequently observed. In some cases coexpression of BRN3A was observed with GFP in the GCL, but this was not a prominent finding. (Fig 6L) Rhodopsin was expressed in the photoreceptor layers; however any expression of GFP could not be differentiated at the photoreceptor layer due to cross-reaction (Fig 6M, 6N, 6O and 6P).

#### Functional results: electrophysiology

The mean blood glucose levels of *Diabetic* group and the mean blood glucose levels of these rats after right eye BMSC, left eye Sham treatment (4^th^ month) are given in Fig 2.

**Fig 7:**
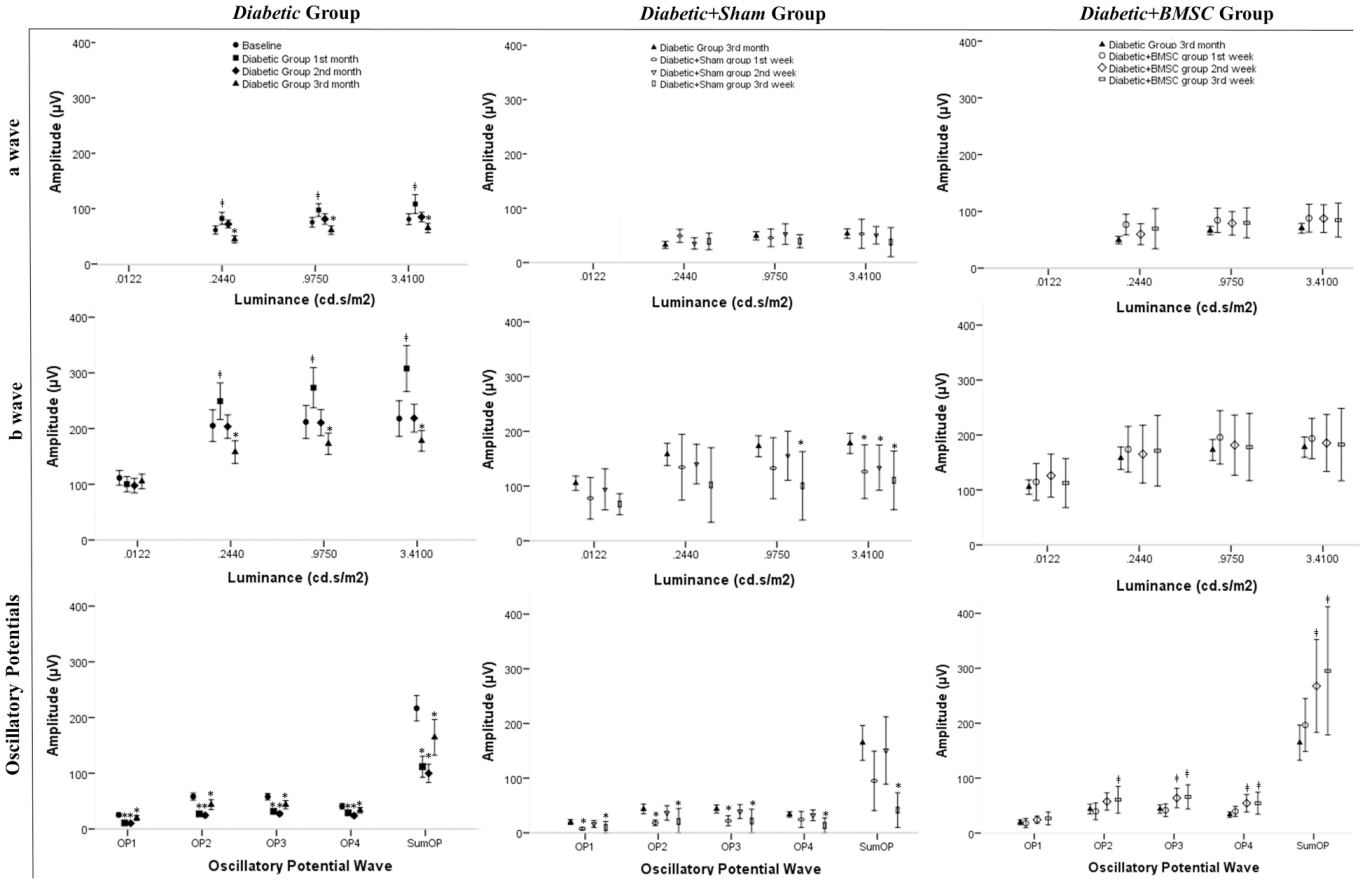
The graph representing the ERG amplitudes of *Diabetic* group at 1^st^, 2^nd^ and 3^rd^ months compared to baseline and *Diabetic+Sham* and *Diabetic+BMSC* groups at 1^st^, 2^nd^ and 3rd weeks of injection compared to the *Diabetic* group at 3^rd^ month of induced diabetes. ǂ, significantly more than the mean amplitude of the Baseline Group *, Significantly less than the mean amplitude of the Baseline Group OP1, Oscillatory Potential Wave 1; OP2, Oscillatory Potential Wave 2; OP3, Oscillatory Potential Wave 3; OP4, Oscillatory Potential Wave 4. SUMOP, The sum of all OP waves at measurement.

The amplitudes of the *Diabetic* group at 1^st^, 2^nd^ and 3^rd^ month in comparison with the *Baseline group* are shown in Fig 7 at the first column. All “a” and “b” wave amplitudes of the *Diabetic* group at 1^st^ month were significantly higher compared to the *Baseline* group, except for the “b” wave obtained at the dim light (“a” wave: *P* = 0.036, *P* = 0.016, *P* = 0.003; “b” *P*>0.05, *P*>0.05, *P* = 0.016, *P*<0.001, respective to increasing light intensity). The “a” wave amplitudes of the *Diabetic* group at 3^rd^ month of diabetes were significantly lower than the *Baseline* group at all light intensities (*P*<0.001, *P* = 0.007, *P* = 0.005, respective to increasing light intensity), and all “b” wave amplitudes were significantly lower except the “b” wave taken at the dim light. (*P*>0.05, *P* = 0.008, *P* = 0.039, *P* = 0.025, respective to increasing light intensity) All OP amplitudes of the *Diabetic* group at 1^st^, 2^nd^ and 3^rd^ month were significantly lower compared to the *Baseline* group (1^st^ and 2^nd^ month, all OP waves P<0.001, 3^rd^ month OP1: P = 0.005, OP4: P = 0.008, other OP waves P<0.001).

The amplitudes of *Diabetic+Sham group* at 1^st^, 2^nd^ and 3^rd^ week compared to *Diabetic group* at 3^rd^ month are shown also in Fig 7 at the second column. The “a” wave amplitudes of the *Diabetic+Sham* group were not significantly different than the *Diabetic* group at 3^rd^ month except the “a” wave on the first week taken at dim light which was higher (*P* = 0.025). The “b” wave amplitudes of *Diabetic+Sham* group were not significantly different from the *Diabetic* group (3^rd^ month) in ERG measurements taken at lower light intensities but were mostly significantly lower in higher light intensities. (1^st^, 2^nd^ and 3^rd^ week at 3.41 cd.s/m^2^ *P* = 0.041; *P* = 0.021 *P* = 0.021 respectively and 3^rd^ week at 0.975 cd.s/m^2^ *P* = 0.021). All OP wave amplitudes were significantly lower at 3^rd^ week of sham injection compared to the *Diabetic* group at 3^rd^ month of induced diabetes. (OP1, OP2, OP3, OP4, and SumOP; *P* = 0.046, *P* = 0.027, *P* = 0.023, *P* = 0.006, and *P*<0.001).

The amplitudes of *Diabetic+BMSC group* at 1^st^, 2^nd^ and 3^rd^ weeks compared to *Diabetic group* at 3^rd^ month are shown in Fig 7 at the third column. None of the “a” or “b” wave amplitudes were significantly different than the *Diabetic* group, except the “a” wave which was higher at first week (*P* = 0.002), but most OP amplitudes were significantly higher (2^nd^ week: O2, O3, O4, and SumOP, *P* = 0.048, *P* = 0.019, *P* = 0.012, *P* = 0.015; at 3^rd^ week O3, O4, and SumOP, *P* = 0.023, *P* = 0.008, and *P* = 0.006, respectively). When the amplitudes of *Diabetic+Sham* and *Diabetic+BMSC* groups were compared with each other, no significant difference in “a” and “b” waves was detected (*P*>0.05, all), but most OP amplitudes were significantly higher in the *Diabetic+BMSC* group (1^st^ week: OP1, OP2, OP3, and SumOP; *P* = 0.026, *P* = 0.017, *P* = 0.011, and *P* = 0.017 respectively. 2^nd^ week: OP1, OP2, OP3, OP4, and SumOP; *P* = 0.044, *P* = 0.029, *P* = 0.029, *P* = 0.034, and *P* = 0.044, respectively. 3^rd^ week: O1, O2, O3, and SumOP; *P* = 0.032, *P* = 0.016, P = 0.016, *P* = 0.08 and *P* = 0.08 respectively).

When *Baseline*, *Diabetic+BMSC*, *Healthy+BMSC* and *Healthy+Sham* groups were compared to each other none of the amplitudes were significantly different to each other with the Kruskal Wallis test (*P*>.05). When the *Diabetic+Sham* group was added to the comparison, all amplitudes were significantly lower in *Diabetic+Sham* group, except the “a” wave amplitudes measured under dim light conditions. Comparing the amplitudes of *Healthy+Sham* or *Healthy+BMSC* to the *Baseline* group separately, none of the ERG waveforms had a significantly different mean values. (Table 1)

**Table 1:** Table to compare the mean amplitudes of all groups.

Light Intensity (cd.s/m^2^)	Wave Type	Baseline Group	Diabetic Group (3^rd^ month)	Diabetic+BMSC Group (3^rd^ week)	Diabetic+Sham Group (3^rd^ week)	Healthy+BMSC Group (3^rd^ week)	Healthy+Sham Group (3^rd^ week)
.0122	a (μV)	111.5 ± 44.2	105.2±48.4	112.5±35.9	66.9±15.4*	147.3±31.5*	137.5±54.4
.244	a (μV)	-50.9±24.6	-33.9±24.3*	-54.1±28.5	-40.5±12.3	-59.1±4.7	-45.8±38.5
	b (μV)	205.3±95.5	157.8±74.6	171.4±51.7	102.1±54.9*	212.7±41.7	201.6±103.1
.975	a (μV)	-64.9±29.7	-50.6±27.7*	-64.2±21.3	-40.8±9.8*	-77.8±25.6	-68.4±28.5
	b (μV)	211.8±99.7	172.8±70.6*	178.0±49.3	100.5±50.4*	223.7±55.5	215.7±90.0
3.41	a (μV)	-70.5±33.1	-54.7±31.4*	-69.2±24.0	-39.2±21.6*	-83.6±22.7	-66.5±48.9
	b (μV)	218.0±107.0	178.0±68.0*	182.6±53.1	110.4±43.1*	216.7±49.8	194.0±96.6
	OP1 (μV)	-23.7±13.7	-19.7±16.2*	-26.7±9.5	-9.5±9.1*	-35.8±15.1	-29.4±16.3
	OP2 (μV)	56.7±25.4	43.5±32.8*	60.5±19.7	20.8±19.2*	74.3±31.4	64.9±31.1
	OP3 (μV)	-58.0±20.1	-43.6±27.7*	-65.7±17.7	-21.5±17.5*	-66.2±25.7	-66.4±24.6
	OP4 (μV)	40.8±17.0	33.6±18.2*	54.3±16.1*	13.3±11.1*	46.9±19.6	53.3±22.3
	SumOP (μV)	216.7±76.6	164.5±117.3*	295.3±94.0	41.4±25.7*	303.5±114.3	291.0±133.6

*, Significantly different compared to the *Basline* group

**Fig 8:**
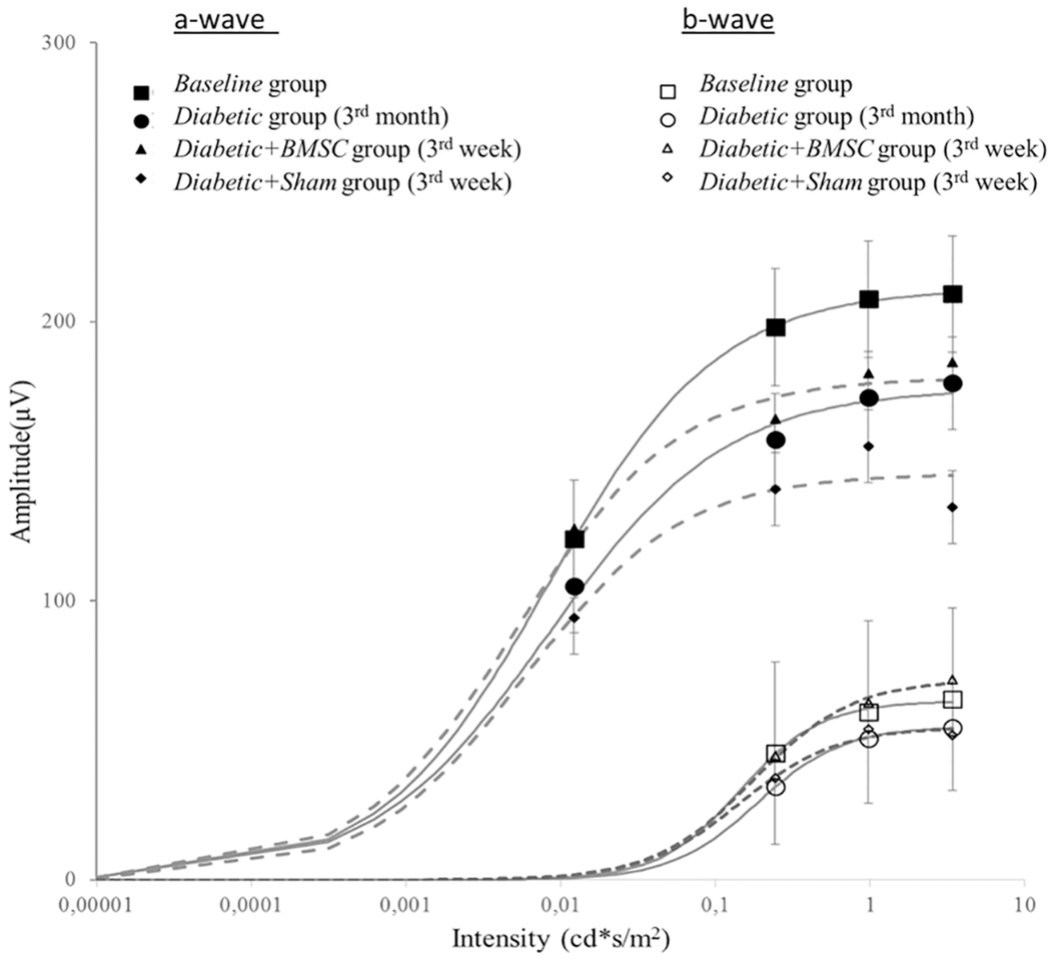
The representative mean intensity-response function of the “a” and “b” waves of the Baseline group, *Diabetic* group (at 3^rd^ month), *Diabetic+BMSC* and *Diabetic+Sham* groups (at 3^rd^ week). ǂ, significantly more than the mean amplitude of the Baseline Group; BMSC, Bone marrow derived mesenchymal stem cells. OP1, Oscillatory Potential Wave 1; OP2, Oscillatory Potential Wave 2; OP3, Oscillatory Potential Wave 3; OP4, Oscillatory Potential Wave 4. SUMOP, The sum of all OP waves at measurement.

**Fig 9:**
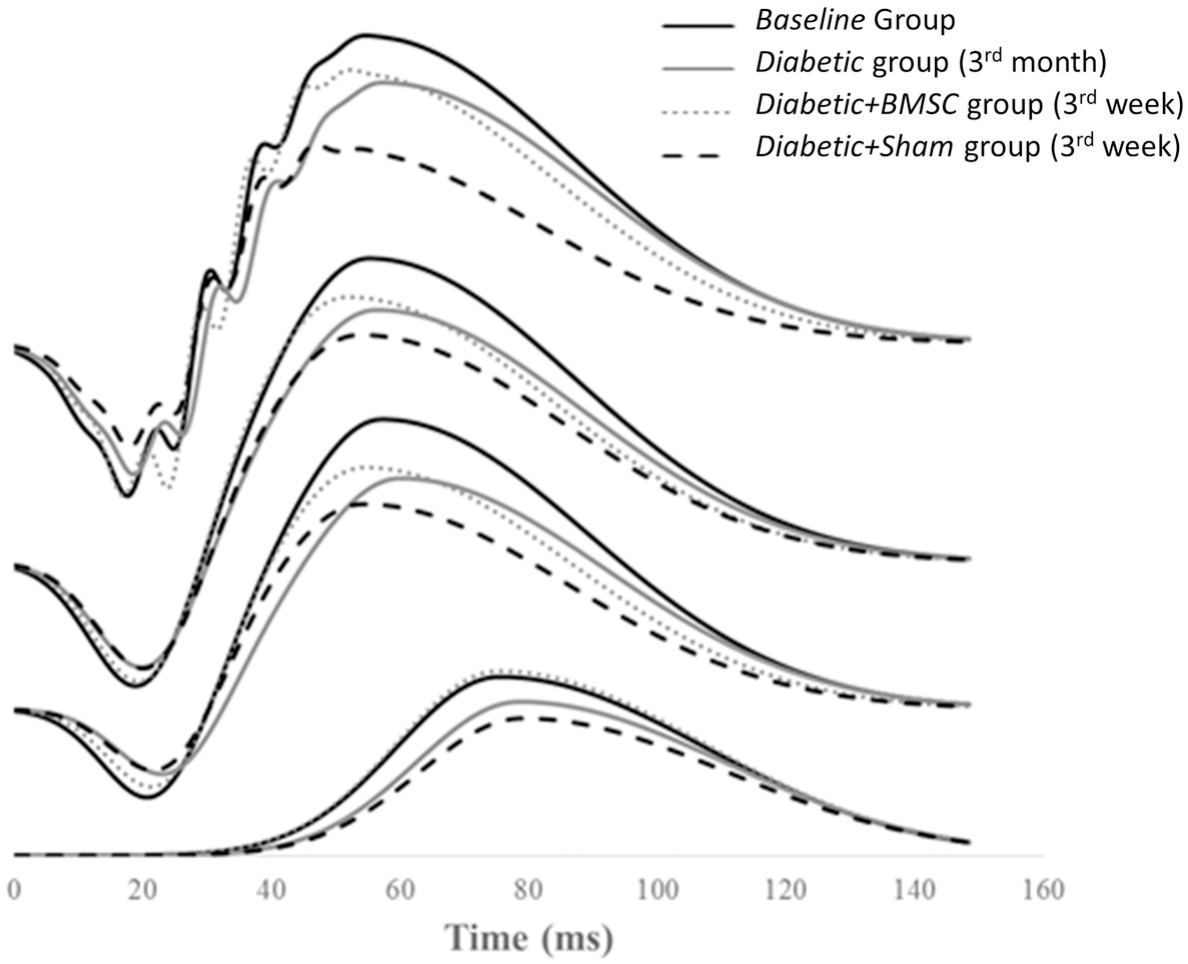
The plotted mean ERG waveforms at increasing light intensities of the *Baseline* group, *Diabetic* group (at 3^rd^ month), *Diabetic+BMSC* and *Diabetic+Sham* groups (3^rd^ week). BMSC, Bone marrow derived mesenchymal stem cells. Sham, in this study 2 μL of balanced salt solution was injected intravitreally as Sham.

**Fig 10:**
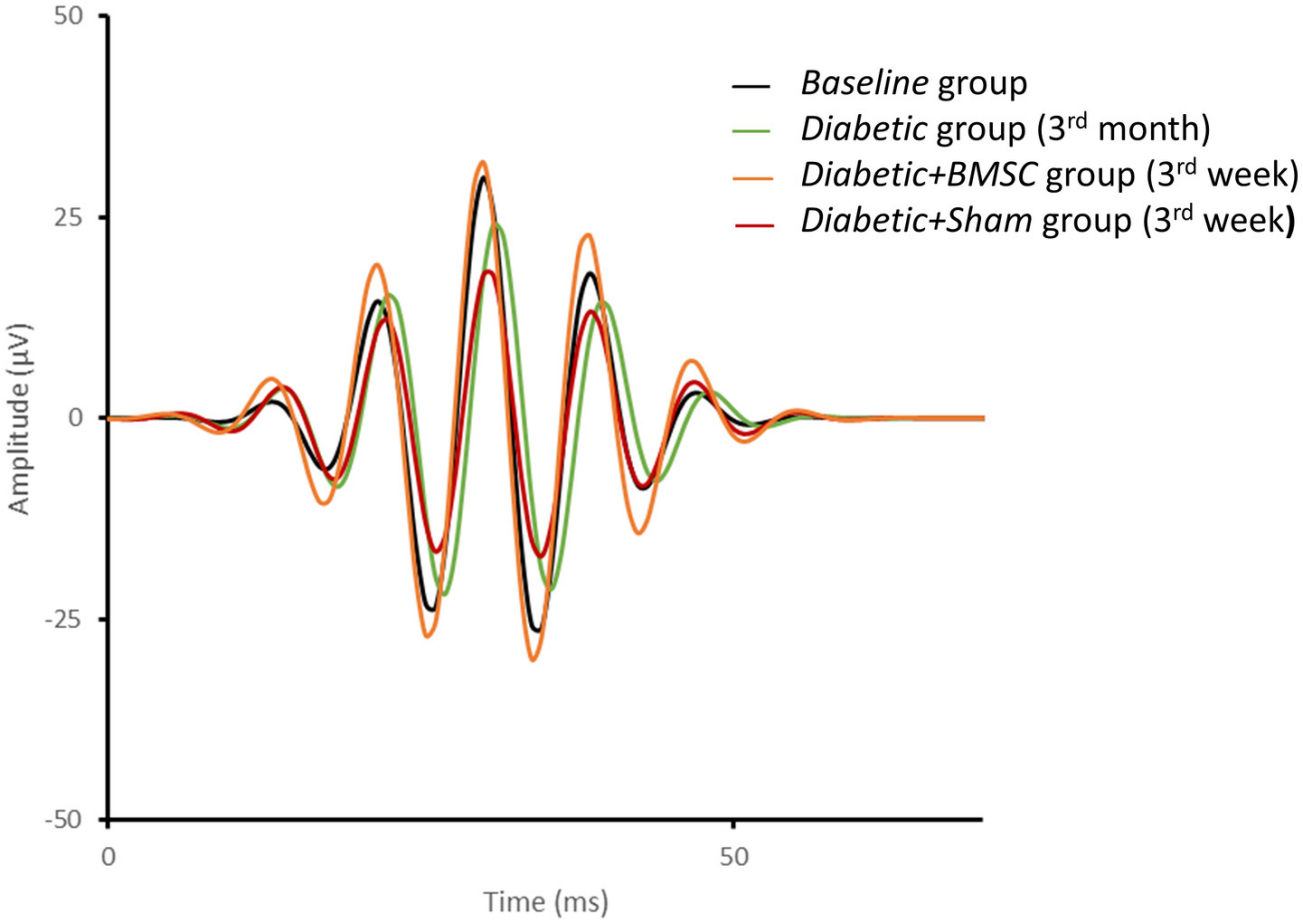
The representative mean OP waveforms of the *Baseline* group, *Diabetic* group (3^rd^ month), *Diabetic+BMSC* and *Diabetic+Sham* groups (3^rd^ week). BMSC; Bone marrow derived mesenchymal stem cells, Sham; in this study 2 μL of balanced salt solution was intravitreally injected as Sham.

The representative mean intensity-response function of the “a” and “b” waves of the *Baseline* group, *Diabetic* group (at 3^rd^ month), *Diabetic+BMSC* and *Diabetic+Sham* treated groups (at 3^rd^ week) are shown in Fig 8 and the representative mean ERG of these groups in four different light intensities are given in Fig 9. Fig 10 shows the representative mean plot of the OP waveforms of the main groups. Compared to the *Control* group the maximum response (Rmax) of b waves were markedly reduced in Diabetic group. (233.7 vs 182.4 μV, respectively). The Rmax values of *Diabetic+Sham group* were lower compared to the *Diabetic+BMSC* group (193.9 vs 156.0 μV). The calculated K constants of the best fit wave were found to be as follows: *Control* group: 0.011; *Diabetic* group: 0.015; *Diabetic+BMSC*: 0.014; *Diabetic+Sham*: 0.018.

The implicit times of main groups are given in the Table 2. *Diabetic* group (3^rd^ month) had prolonged implicit times compared to the *Baseline* group. The *Diabetic+Sham* and *Diabetic +BMSC* groups had no statistically significantly different implicit times in any of the ERG components, compared to the *Diabetic* group, whereas the *Healthy+Sham* and *Healthy+BMSC* groups did not have significantly different implicit times compared to the *Baseline* group in any of the waveforms.

**Table 2:** The implicit times of ERG components in all main groups. The implicit times of “a” and “oscillatory potential” waves were significantly prolonged in the *Diabetic* group at 3^rd^ month of induction of diabetes. *Diabetic+sham* or *Diabetic+ BMSC* groups did not have significantly different values compared to diabetic group. On the other hand *Healthy+Sham* and *Healthy+BMSC* groups did not have significantly different values compared to the *Baseline* group.

Light Intensity (cd.s/m^2^)	Wave Type	Baseline Group	Diabetic Group (3^rd^ month)	Diabetic+BMSCgroup (3^rd^ week)	Diabetic+Sham group (3^rd^ week)	Healthy+BMSC group	Healthy+Sham group
.0122	b (ms)	77.9±11.6	81.3±14.2	74.5±11.0	77.7±11.9	77.5±3.5	77.2±4.4
.244	a (ms)	22.2±1.6	25.2±9.4^ǂ^	24.6±.8	26.9±3.4*	21.9±1.0	22.5±1.4
	b (ms)	58.4±10.9	61.2±15.8	54.6±6.9	54.5±7.3	50.8±5.7	50.8±2.7
.975	a (ms)	20.5±1.7	21.7±3.0^ǂ^	21.4±.9	23.6±2.6	19.6±1.4	19.8±1.1
	b (ms)	55.4±13.1	57.1±10.7	49.1±4.3	51.4±7.1	47.5±5.4	49.5±3.3
3.41	a (ms)	19.0±2.2	20.6±3.1^ǂ^	17.7±2.1*	21.3±2.7	19.4±1.2	19.8±.7
	b (ms)	53.6±14.0	57.1±11.2	52.9±12.3	57.6±10.8	46.8±5.4	48.3±6.1
	OP1 (ms)	26.3±3.1	27.8±5.3	28.4±3.7	29.8±3.8	26.3±1.1	26.9±1.2
	OP2 (ms)	30.3±3.1	32.1±5.3	32.9±3.7	36.6±4.5*	30.1±.9	30.9±1.5
	OP3 (ms)	34.3±3.1	36.4±5.6	37.3±4.0	40.8±4.7*	34.3±1.0	34.7±1.7
	OP4 (ms)	38.9±3.3	41.3±5.9	42.0±4.5	45.1±5.0	39.3±.9	39.3±1.6
	SumOP (ms)	129.8±12.4	137.5±22.0	140.6±15.9	152.3±15.2*	129.9±3.7	131.8±5.7

^ǂ^, significantly different compared to the *Baseline* group (Mann Whitney U test).

*, significantly different compared to the *Diabetic* group at 3^rd^ month (Mann Whitney U test).

Please note that the *Diabetic* group and *Healthy* groups are compared to the Baseline group. *Diabetic+BMSC* and *Diabetic+Sham* groups are compared to the *Diabetic* group at 3^rd^ month.

The paired analysis of 9 animals for ERG values before and one week after injection of BMSC or sham revealed that the O1, O2, and O3 waves significantly decreased in the sham-injected eyes left eyes (*Diabetic+Sham* group) at the end of 1 week (P = 0.044, P = 0.011, and P = 0.022, respectively), while in the BMSC-injected right eyes (*Diabetic+BMSC* group), the O1, O2, and O3 waves did not change (P>0.05), the O4 wave increased significantly (P = 0.035).

At the end of 3 months of STZ induced diabetes, the ERG amplitudes of eyes (n = 38) from diabetic rats that had received multiple anesthesia were compared to those who received only one anesthesia (n = 16) and there was no significant difference between the groups in any of the amplitudes (p>0.05).

## Discussion

Müller cells normally respond following activation of the intermediate filament protein, which leads to GFAP hypertrophy; this is known as reactive gliosis.[39–41] In the early stages of STZ induced diabetes, the neuronal and glial alterations in the retina precede the typical vascular changes. It is known that Müller cells cultured in a high-glucose medium gradually increase GFAP expression. [42] Oxidative stress is also known to increase the GFAP expression in diabetic Müller cells.[43, 44] Normally after about 6 weeks of induction of diabetes, Müller cell gliosis and neuronal deficits begin to become prominent. [45] Glutamine synthase, vimentin and GFAP are biomarkers of Müller cells. [46, 47] Overexpression of GFAP and vimentin occurs during Müller cell gliosis, which is known to be increased in diabetic retinopathy or STZ induced diabetic retinopathy [39, 46, 48] The results in the present study confirmed the increased retinal gliosis in the *Diabetic* group, (Fig 5) as both vimentin and GFAP expression was found to be increased in in the *Diabetic* groups compared to *Healthy* groups.

In the immunofluorescence analysis of the present study GFP-labelled BMSC were detectable in the retina and were double stained with anti-GFAP and anti-vimentin, suggesting the differentiation of BMSC into retinal glial cells. Apparently the integration occurred mainly in the diabetic eyes and also scarcely in the healthy eyes. (Fig 6)

The finding that the expression of vimentin was found less in *Diabetic+BMSC* group compared to *Diabetic+Sham* group suggests a protective effect of BMSC against gliosis during the 3 weeks after intravitreal injection. (Fig 5) Intravitreally injected BMSC are recently shown to induce a graft induced reactive gliosis in healthy rats in the long term. Contrary to this study our short term results showed a decrease in gliosis. Previously it was demonstrated that stem cells have protective effects against retinal vasculopathy by preventing capillary loss and retinal capillary dropout. [11, 12, 43, 44] These cells are especially important in the formation of physiological vessels, rather than in pathological angiogenesis. [49] In a STZ-induced rodent model of diabetic retinopathy, BMSC were shown to improve the integrity of the blood–retina barrier. [17] BMSC can also selectively target gliosis, and provide neurotrophic effects. [50, 51] It has also been demonstrated that BMSC can increase the retinal and intravitreal concentrations of neuroprotective growth factors. [52]

In this study the ERG investigation of STZ induced diabetic rats aimed to investigate the functional effects of stem cells on the retina. In previous studies on animals the most common ERG finding of diabetes is reduced amplitude and prolonged implicit time of OP and in some studies an altered “a” and “b” waveforms. [53–55] In our results we observed an increase in the mean “a” and “b” wave amplitudes at 4th week of diabetes. Our literature search revealed a study [29] where a paradoxical increase in the photoreceptor response at 8^th^ week was observed and this later returned to control levels at 11^th^ week. This finding was described as a chance observation. Wong et al. [56] on the other hand have found that at 4th week of STZ induced diabetes, “b” wave was observed to be increased. According to their modelling, a combination of a delay in PII, slow PIII and a reduction in the slow PIII would be adequate to account for the “b” wave changes. They interpreted these changes to be related with the dysfunction of Muller cells (delay and reduction in slow-PIII) and bipolar cell dysfunction (PII delay). In the present study, similarly the increased mean “b” wave amplitude may be explained with a delay in PII bipolar cell dysfunction as the “b” wave implicit times were all found to be significantly delayed (p<0.01). A delay of positive PII component may be speculated to increase the “a” wave amplitude, but otherwise could be interpreted as a chance observation.

An increase in the K constant of Naka-Rushton equation indicates a sensitivity reduction. The calculated K values in the best curve fit showed a reduction in sensitivity in *Diabetic* group, and a relative increase in BMSC injected eyes in the present study.

The most common early ERG finding in diabetes is the reduced amplitude and prolonged implicit time in OPs, as well as an altered photoreceptor response. OPs are high-frequency wavelets with small amplitudes, which are observed in the ascending limb of “b” waves. They are thought to involve in amacrine cell activity, [57] and early OP changes reflect the susceptibility of these cells to diabetes. [54] OPs are known to be the most sensitive ERG indicator of DR.[53, 58–62] Reduced amplitudes of OPs were also found to be related to the severity of DR.[63] The results in this study showed a prominent reduction in especially OP wave amplitudes in STZ induced diabetes, beginning as early as in the first month. After the injection of BMSC in the *Diabetic+BMSC* group the amplitudes have been found to be increased in the next 3 weeks, whereas the *Diabetic+Sham* group the amplitudes gradually decreased (Fig 7). As we injected to the right eyes of *Diabetic* group BMSC and left eye same volume of sham on the same day, theoretically the only condition that may lead to significant difference in retinal function can be the presence of BMSC in the right eye injections. Considering that OPs are a good indicator of the disease, the intravitreal injection of BMSC in the *Diabetic+BMSC* group may point to a target-directed treatment, as the greatest change was observed in OP waves.

The precise molecular basis for this electrophysiological effect remains unknown because the specific cells in the retina that are responsible for the generation of OP waves are still being debated.[54] These cells are widely believed to be generated by the activity of the inner retina. Müller cells take part in GABA uptake, which means that they are directly involved in the synaptic activity in the inner retina. [64, 65] It has been shown that the GABA-signaling pathway is disturbed in DR, and that GABA is accumulated in the inner retina which, in turn, may alter OP changes. [55] The integration of GFP labelled BMSC was predominantly observed in the inner retina in the present study.

There are various ongoing clinical trials on the prevention or reversal of disabling vision loss with the help of stem cells. Most of these studies are designed using the intravitreal injection of BMSC. As 2015, there are eight ongoing clinical trials of intravitreal BMSC treatments, where the total subject count has reached 450. [66] Although DR is one of the most important visually debilitating diseases, only one ongoing clinical trial is targeting DR. [67] This may be due to the low number of experimental studies conducted in this specific area. Though several studies have pointed to various beneficial effects of stem cells in DR, [17, 52, 68–70] the present study demonstrates a detailed ERG analysis, specifically an improvement in OP waves.

It is previously shown that diabetes leads to significant loss of Muller cells. [71] Their loss may lead to deterioration in ERG signals, as they have neural progenitor cell properties and take part in the retinal repair. [47, 64] In the present study the coexpression of Vimentin, GFAP and GFP indicates that intravitreally transplanted BMSC may have been differentiated to Muller glia, as Vimentin and GFAP are markers of Muller cells. Previously it is reported that in bFGF+B27-containing differentiation medium, retinal stem cells differentiate into Müller cells. [72] Alternatively embrionic stem cells are shown to release microvesicles that induce dedifferentiation and pluripotency of Muller cells. [73] Therefore we are in opinion that injected BMSC may have been differentiated to Muller glia, or induce the native counterparts.

The power of the study in differentiating the mean OP amplitudes between *Diabetic* and *Baseline* groups was 95%, and the difference between BMSC and sham injected eyes 97% (taken α = 0.05). However the power was low in comparing the effect of BMSC vs Sham among healthy animals, as the subject number of healthy animals was low (n = 6). One of the weak sides of the study is that ideally all subjects should be analyzed in a paired manner during the course of the study, however it would be inappropriate to perform so many anesthesia for each animal. Another weak point is that a confocal microscope was not available for pathology investigations. As indicated in a review about STZ model, there are many variations in the injection protocol in terms of dosage, route of injection, and with or without insulin compensation that are usually based on the practice in individual laboratories. [74] We did not use any additional buffer solutions for diabetic animals during the course of the study as we were unable to find a definitive rule when buffer solutions should be applied in STZ model.

We experienced a high variability in STZ sensitivity in individual rats, as some rats needed a second dose. Although age and gender matched rats were used, the dose that caused severe hyperglycemia in some rats, failed to do so in others and those rats received a second dose. In the literature also STZ sensitivity is reported to be high among different rodent strains and even among individuals. The rats in our cohort were provided by the same supplier, however different generations within a colony may have exacerbated the difference.

We are in opinion that in future studies levels of inflammatory mediators such as IL-1 beta and TNF should be measured, which are increased in Müller cell gliosis, to assess the anti-inflammatory effects of stem cells in the diabetic retina.

## Conclusion

Stem cells have been highlighted as a promising regenerative therapy in retinal diseases. The most commonly used method is the intravitreal injection of BMSC. In this study, we observed integration of BMSC into the retina and to exert possible beneficial effects of the intravitreal injection of BMSC in DR by means of ERG. We have seen a gradual improvement in the most pathognomonic ERG sign of DR: the OP. The BMSC apparently decreased the occurrence of retinal gliosis, and they had also differentiated into retinal glial cells in the inner retina.

## Supporting Information

S1 Fig Anti-GFP antibody immunofluorescence staining images of a retinal section from *Healthy + Sham* group. (JPG)

S2 Fig Anti-GFP antibody immunofluorescence staining images of a retinal section from *Healthy + Sham* group. (JPG)

S3 Fig Anti-GFP antibody immunofluorescence staining images of a retinal section from *Healthy + Sham* group. (JPG)

S4 Fig Anti-GFP antibody immunofluorescence staining images of a retinal section from *Healthy + BMSC* group. (JPG)

S5 Fig Anti-GFP antibody immunofluorescence staining images of a retinal section from *Healthy + BMSC* group. (JPG)

S6 Fig Anti-GFP antibody immunofluorescence staining images of a retinal section from *Healthy + BMSC* group. (JPG)

S7 Fig Anti-GFP antibody immunofluorescence staining images of a retinal section from *Diabetic + Sham* group. (JPG)

S8 Fig Anti-GFP antibody immunofluorescence staining images of a retinal section from *Diabetic + Sham* group. (JPG)

S9 Fig Anti-GFP antibody immunofluorescence staining images of a retinal section from *Diabetic + Sham* group. (JPG)

S10 Fig Anti-GFP antibody immunofluorescence staining images of a retinal section from *Diabetic + BMSC* group. (JPG)

S11 Fig Anti-GFP antibody immunofluorescence staining images of a retinal section from *Diabetic + BMSC* group. (JPG)

S12 Fig Anti-GFP antibody immunofluorescence staining images of a retinal section from *Diabetic + BMSC* group. (JPG)

S13 Fig Anti-Brn3a, Anti-GFP and overlay images of a retinal section from *Diabetic + BMSC* group. (JPG)

S14 Fig Anti-Brn3a, Anti-GFP and overlay images of a retinal section from *Healthy + BMSC* group. (JPG)
