# Correction: Retinal Electrophysiological Effects of Intravitreal Bone Marrow Derived Mesenchymal Stem Cells in Streptozotocin Induced Diabetic Rats

**DOI:** 10.1371/journal.pone.0165219

**Published:** 2016-10-18

**Authors:** Eren Çerman, Tolga Akkoç, Muhsin Eraslan, Özlem Şahin, Selvinaz Özkara, Fugen Vardar Aker, Cansu Subaşı, Erdal Karaöz, Tunç Akkoç

There is an error in affiliation #4 for authors Cansu Subaşı and Erdal Karaöz. Affiliation #4 should be:

Center for Regenerative Medicine and Stem Cell Research & Manufacturing (LivMedCell), Liv Hospital, Istanbul, Turkey.

## References

[1] Çerman E, Akkoç T, Eraslan M, Şahin Ö, Özkara S, Vardar Aker F, et al (2016) Retinal Electrophysiological Effects of Intravitreal Bone Marrow Derived Mesenchymal Stem Cells in Streptozotocin Induced Diabetic Rats. PLoS ONE 11(6): e0156495 doi: 10.1371/journal.pone.0156495 27300133 10.1371/journal.pone.0156495 PMC4907488

