# Supplementary figures and images for: Retinal Electrophysiological Effects of Intravitreal Bone Marrow Derived Mesenchymal Stem Cells in Streptozotocin Induced Diabetic Rats
Source: PLoS One. 2016 Jun 14;11(6):e0156495. doi: 10.1371/journal.pone.0156495 (PMC4907488; doi:10.1371/journal.pone.0156495)

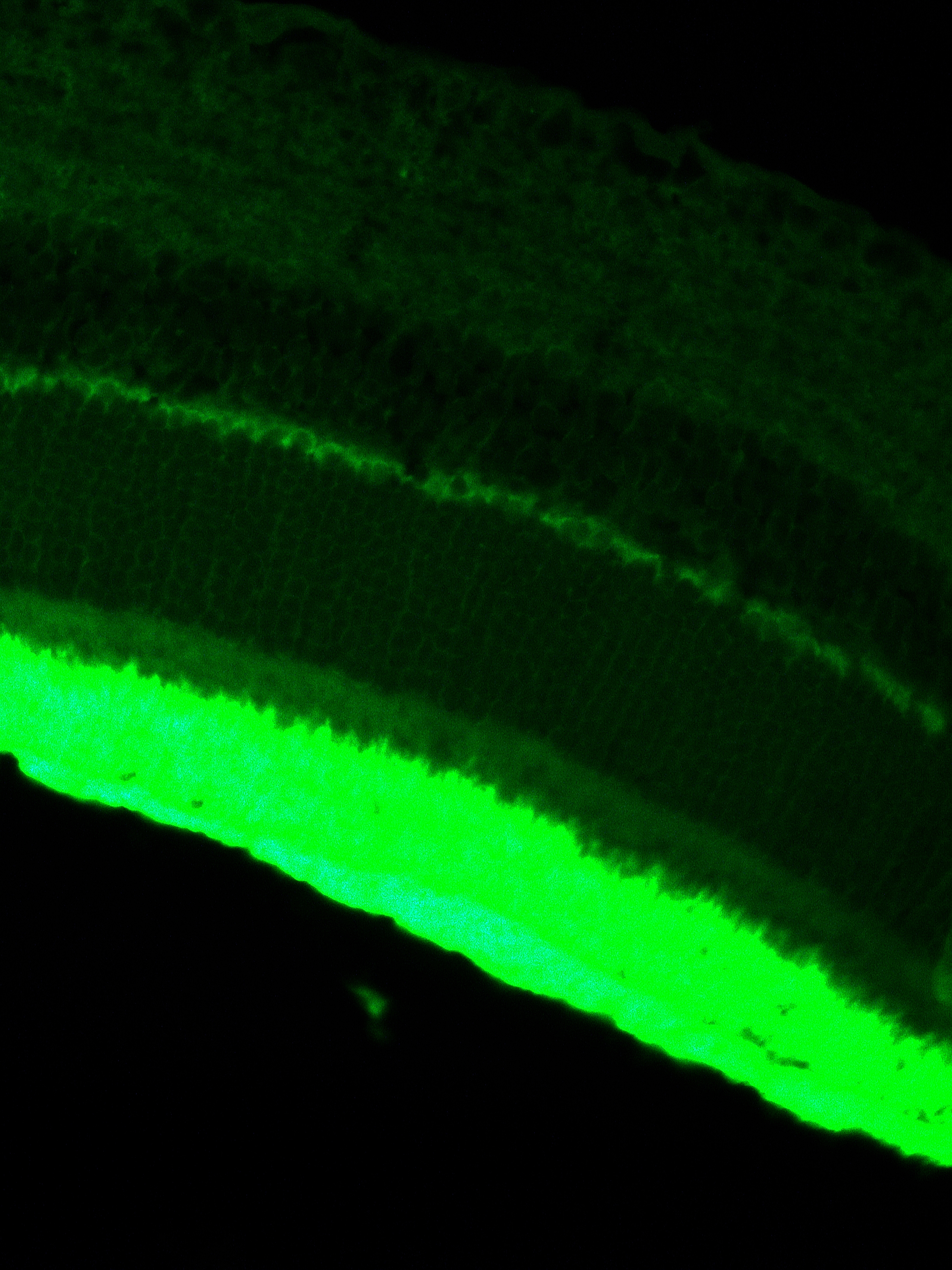

Supplement: S1 Fig — (JPG) [file pone.0156495.s001.jpg]

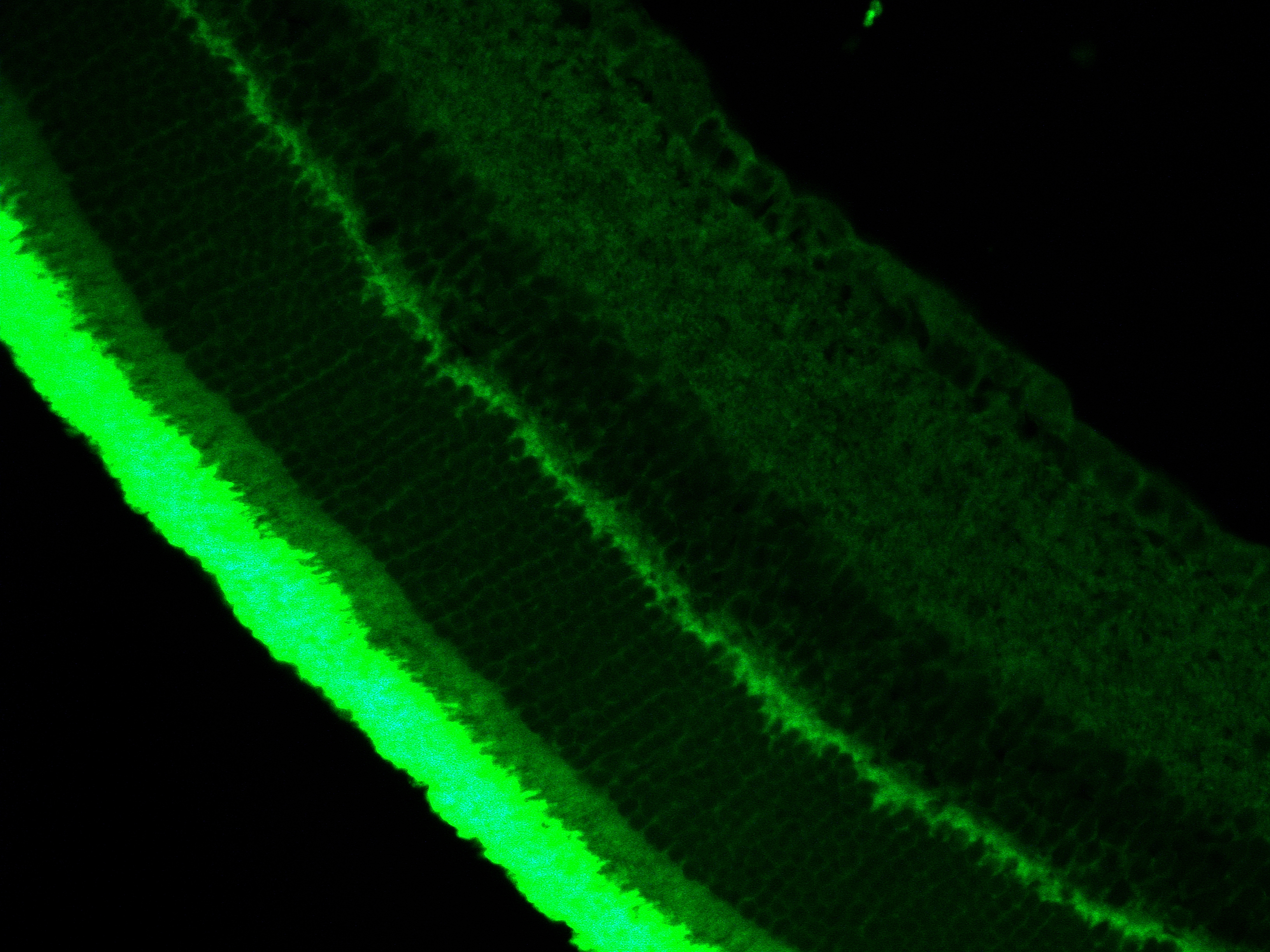

Supplement: S2 Fig — (JPG) [file pone.0156495.s002.jpg]

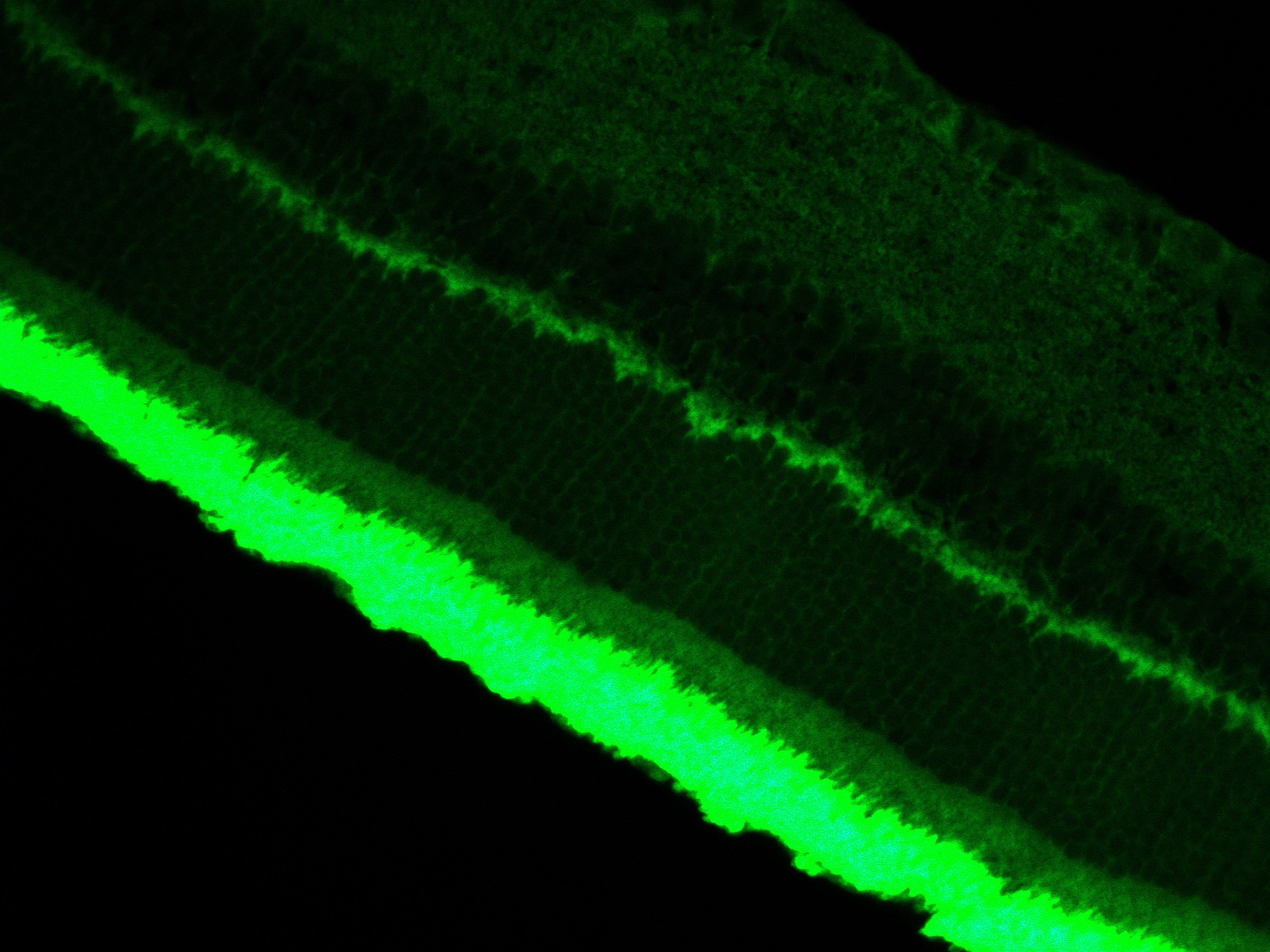

Supplement: S3 Fig — (JPG) [file pone.0156495.s003.jpg]

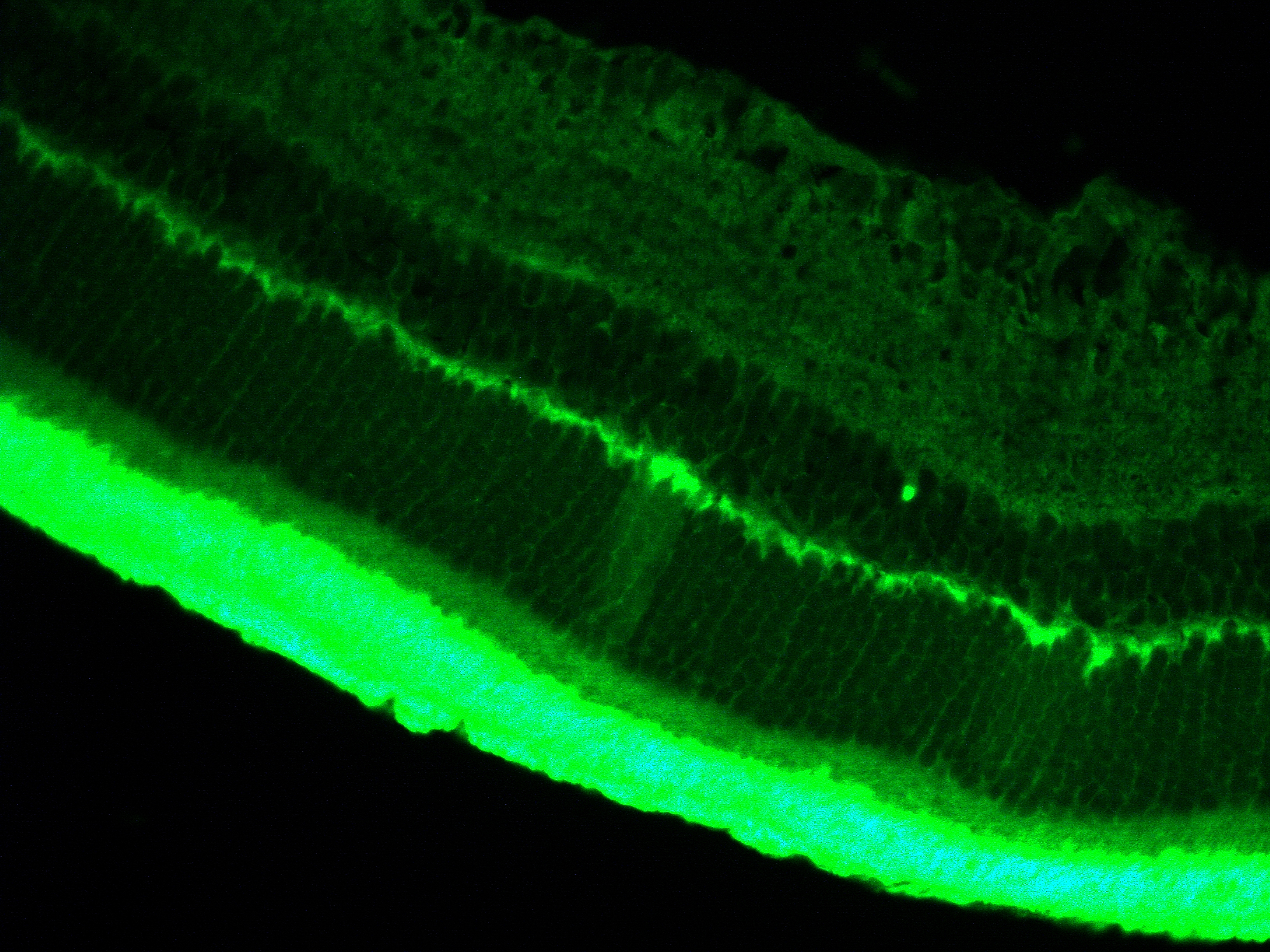

Supplement: S4 Fig — (JPG) [file pone.0156495.s004.jpg]

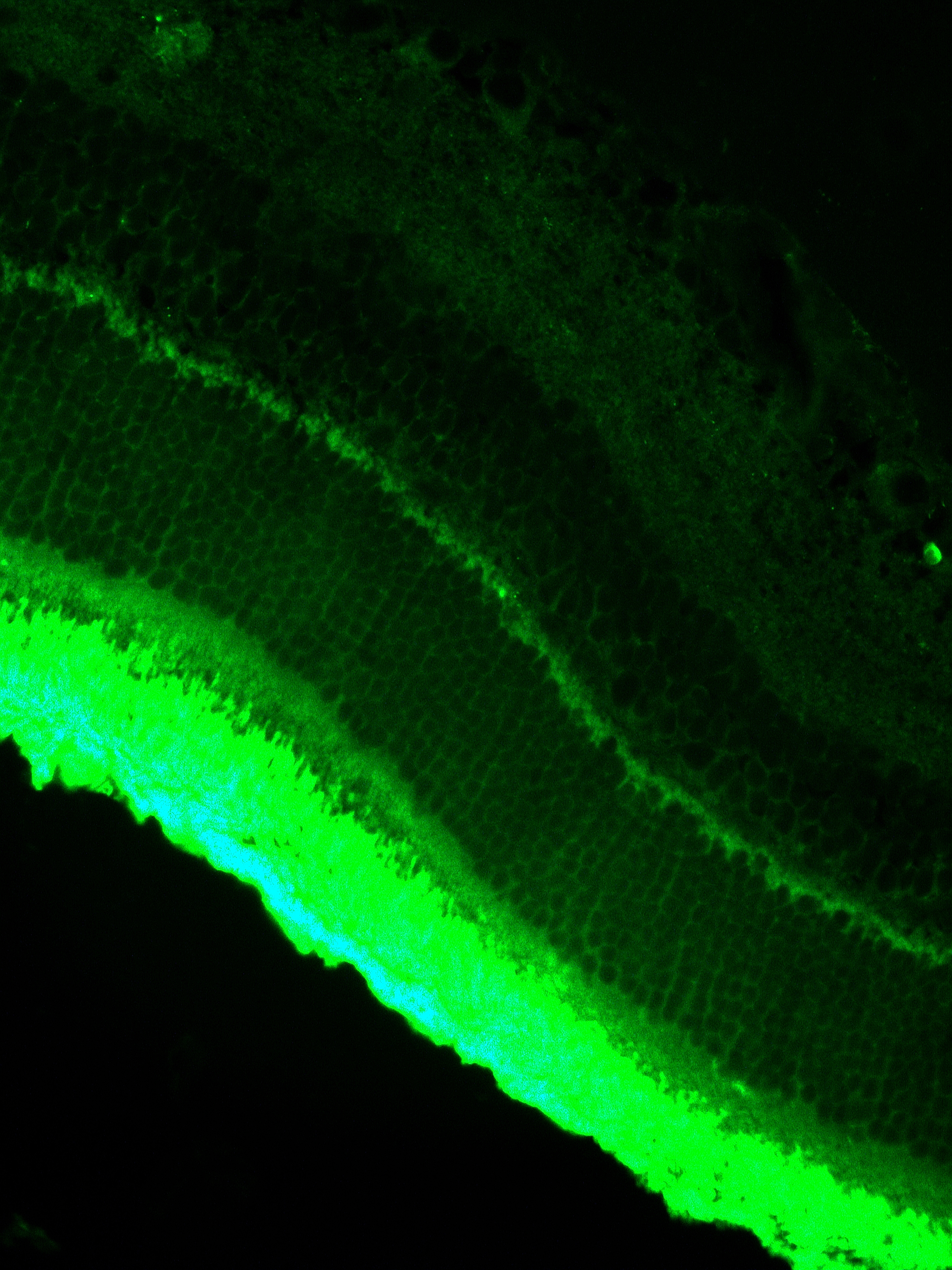

Supplement: S5 Fig — (JPG) [file pone.0156495.s005.jpg]

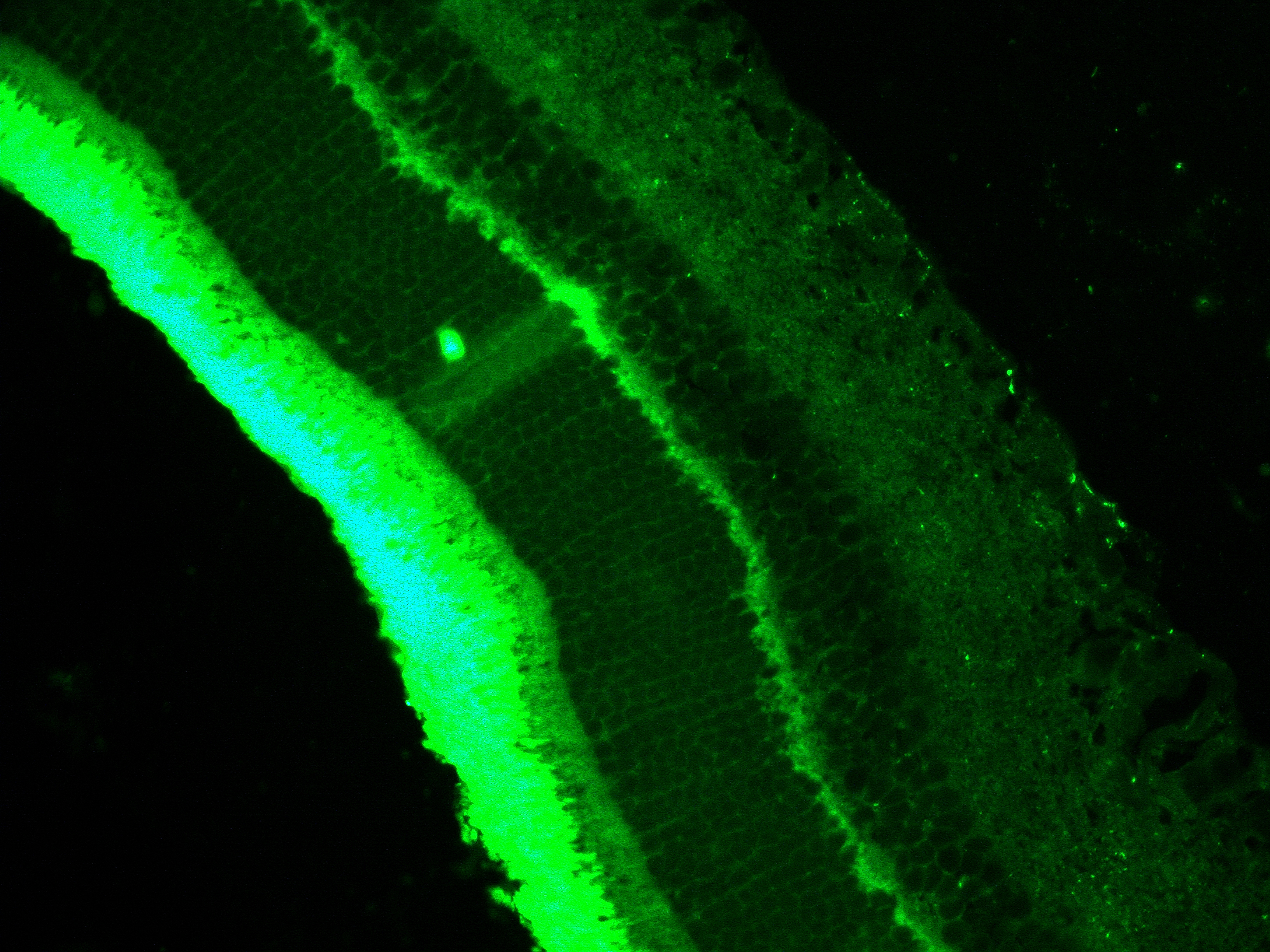

Supplement: S6 Fig — (JPG) [file pone.0156495.s006.jpg]

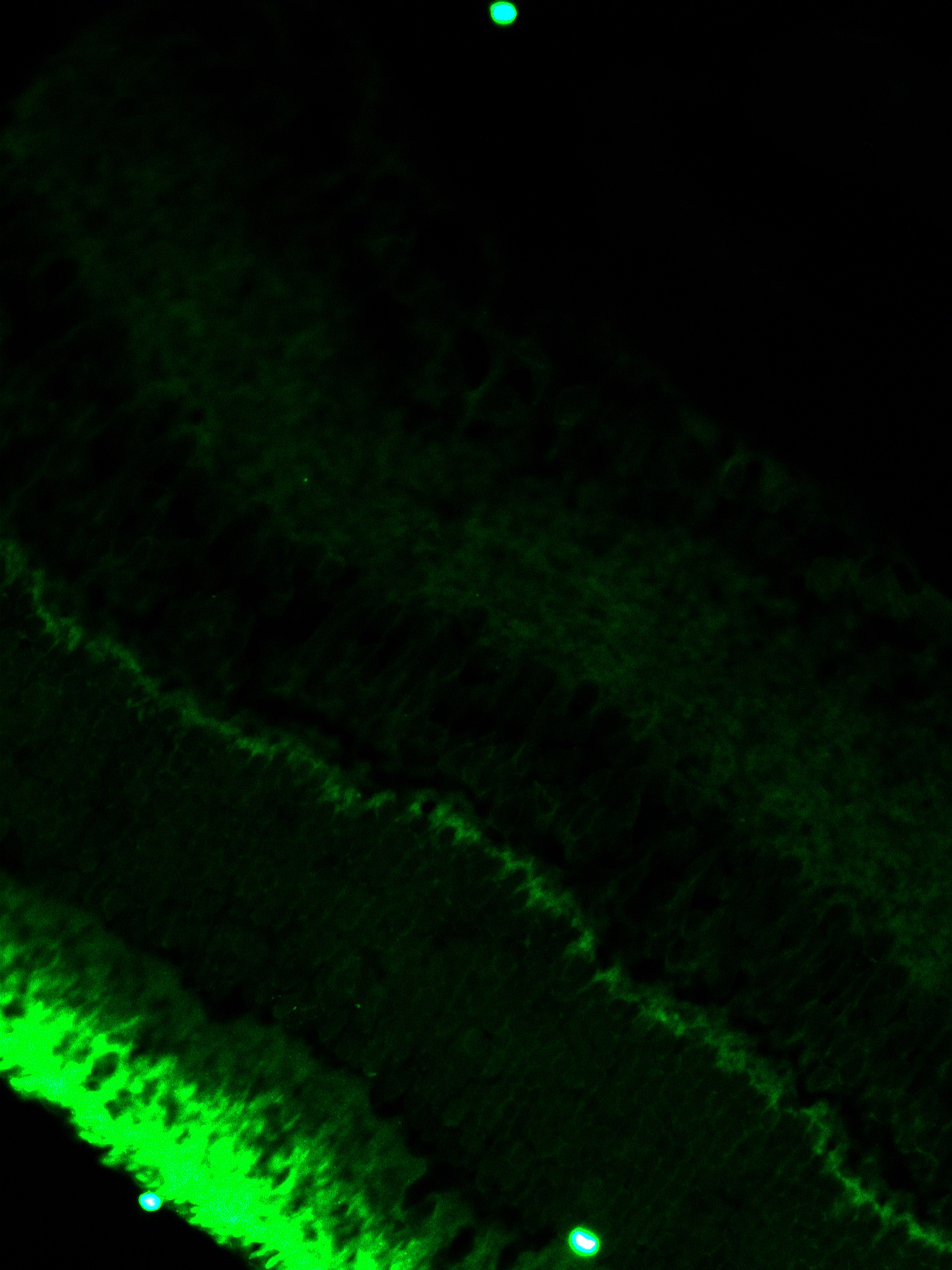

Supplement: S7 Fig — (JPG) [file pone.0156495.s007.jpg]

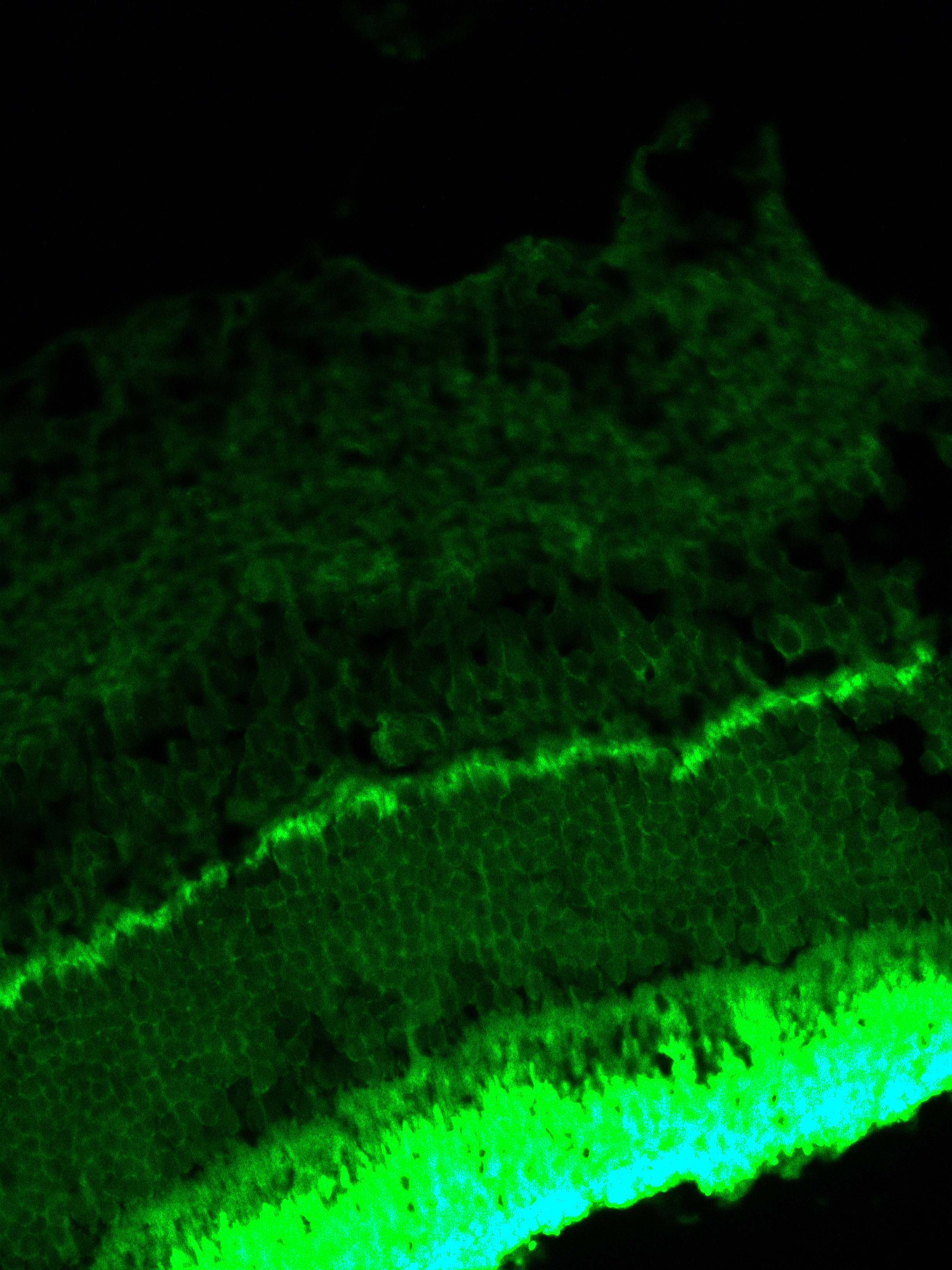

Supplement: S8 Fig — (JPG) [file pone.0156495.s008.jpg]

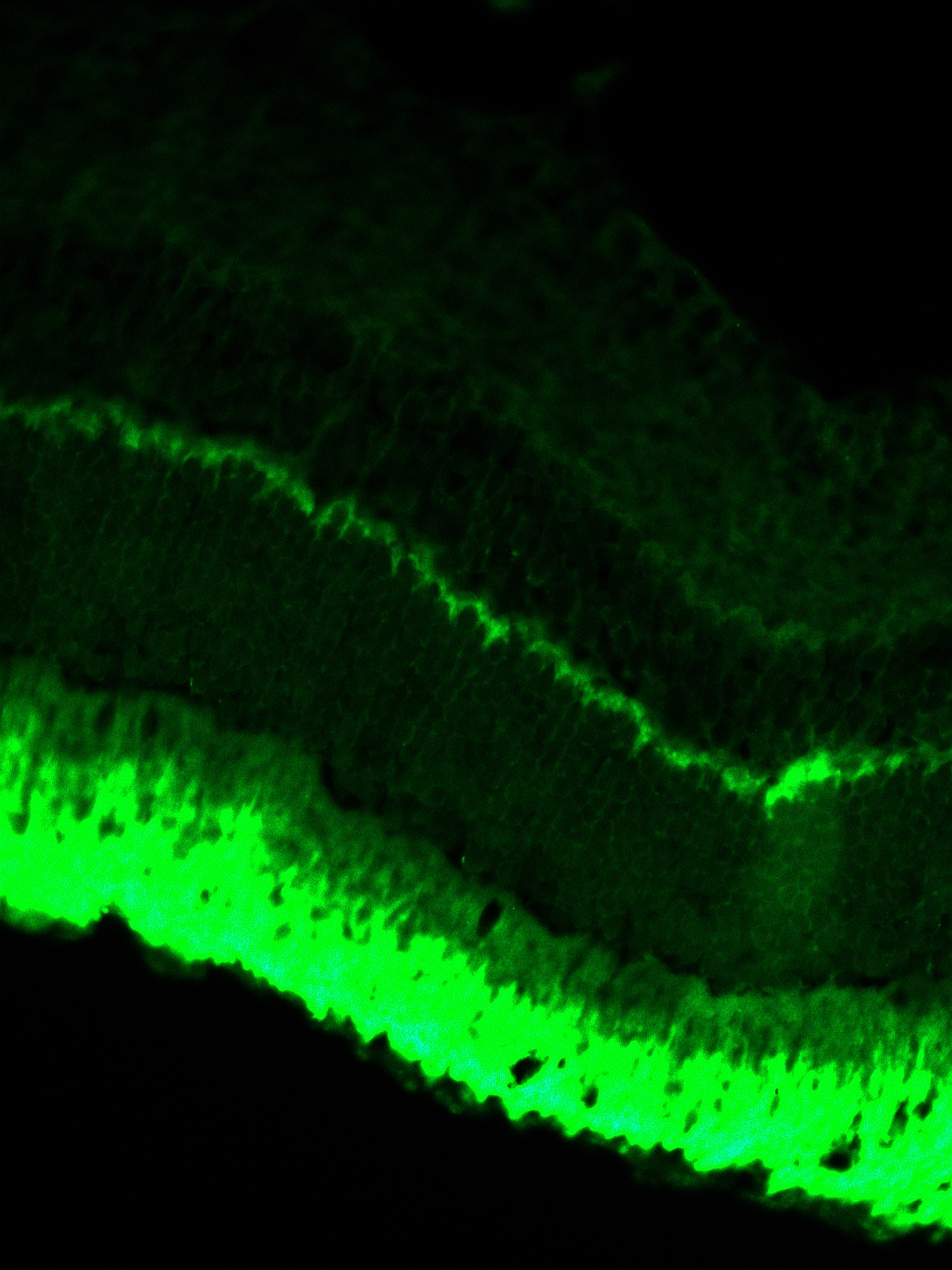

Supplement: S9 Fig — (JPG) [file pone.0156495.s009.jpg]

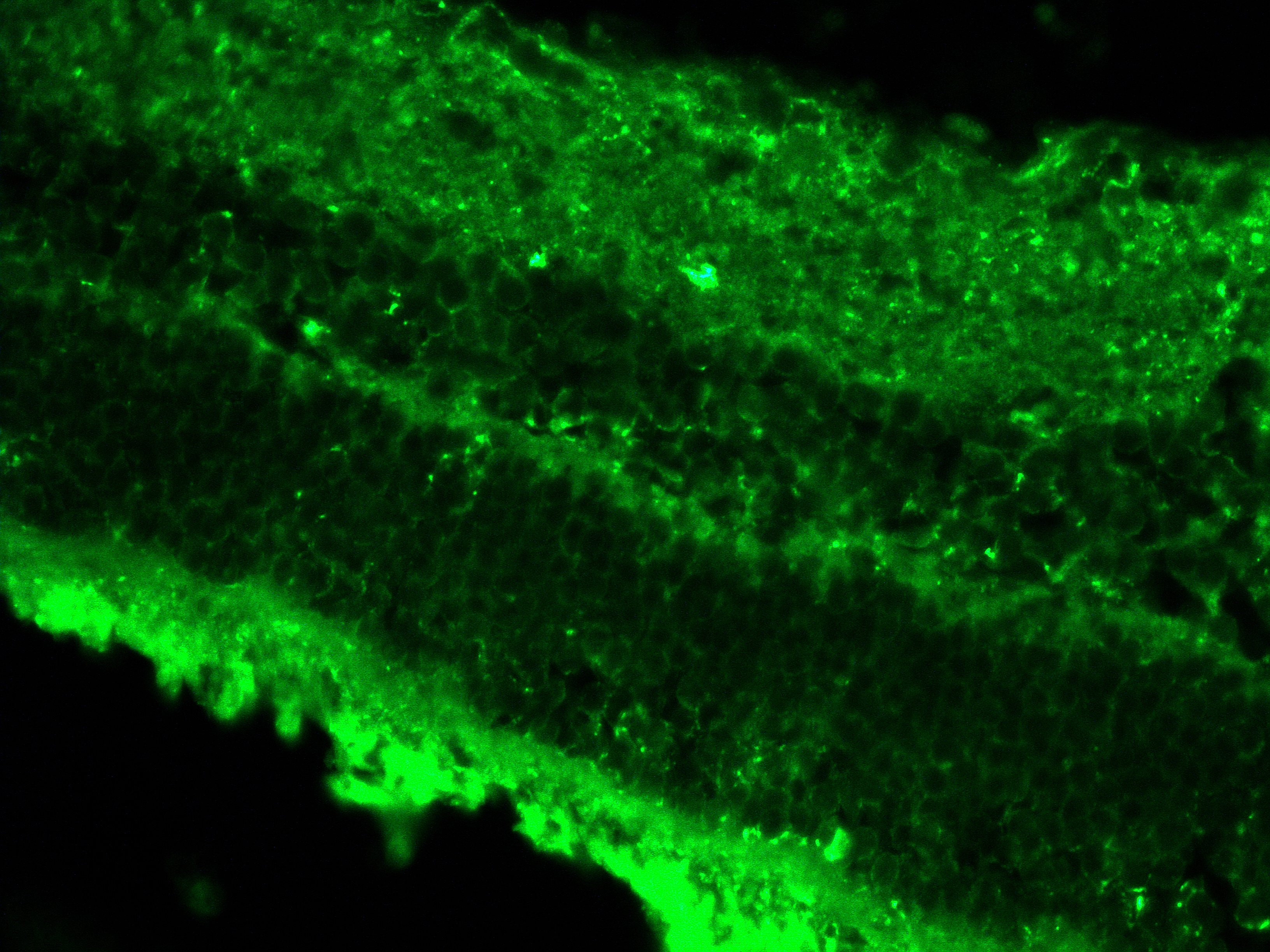

Supplement: S10 Fig — (JPG) [file pone.0156495.s010.jpg]

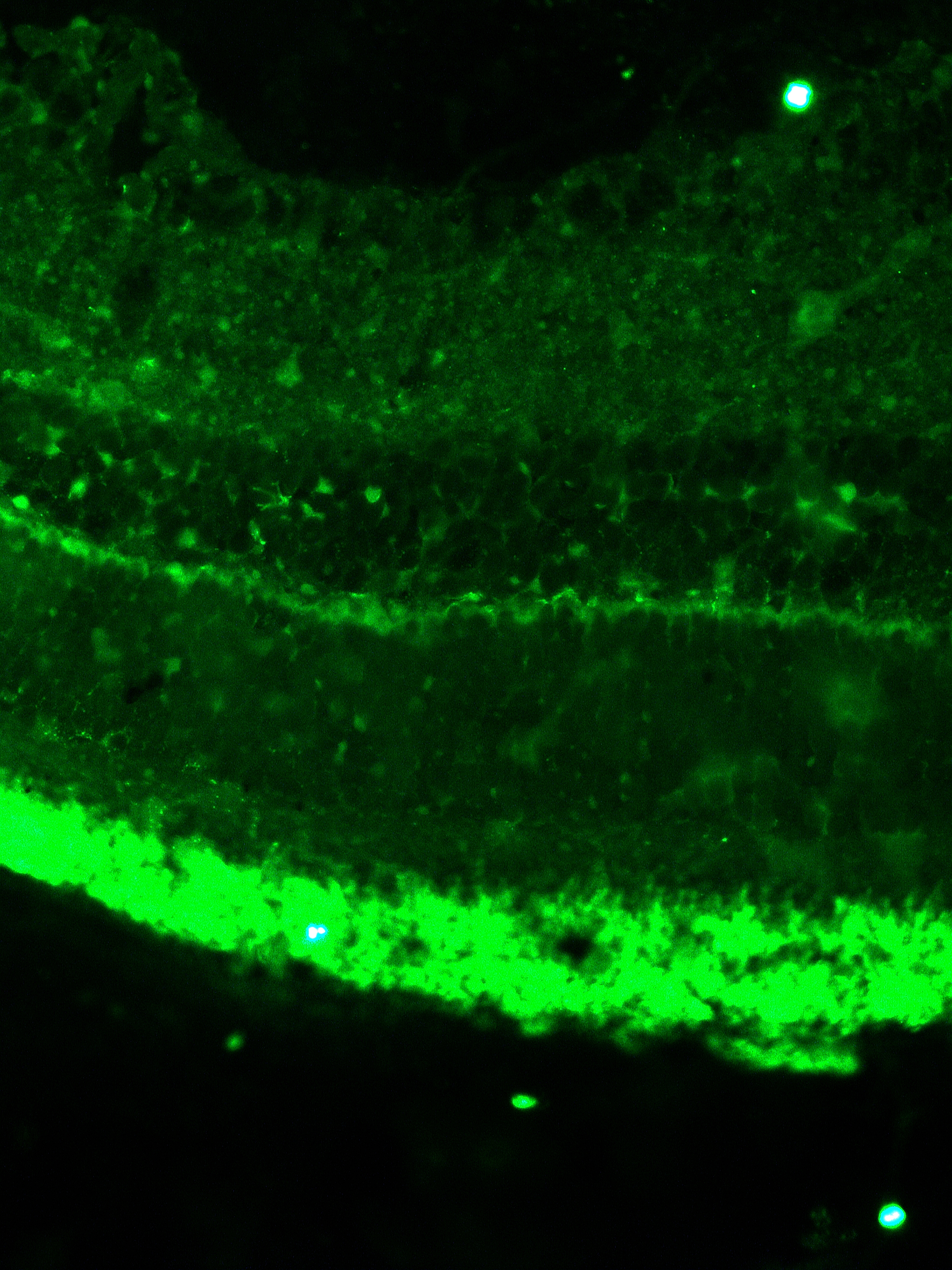

Supplement: S11 Fig — (JPG) [file pone.0156495.s011.jpg]

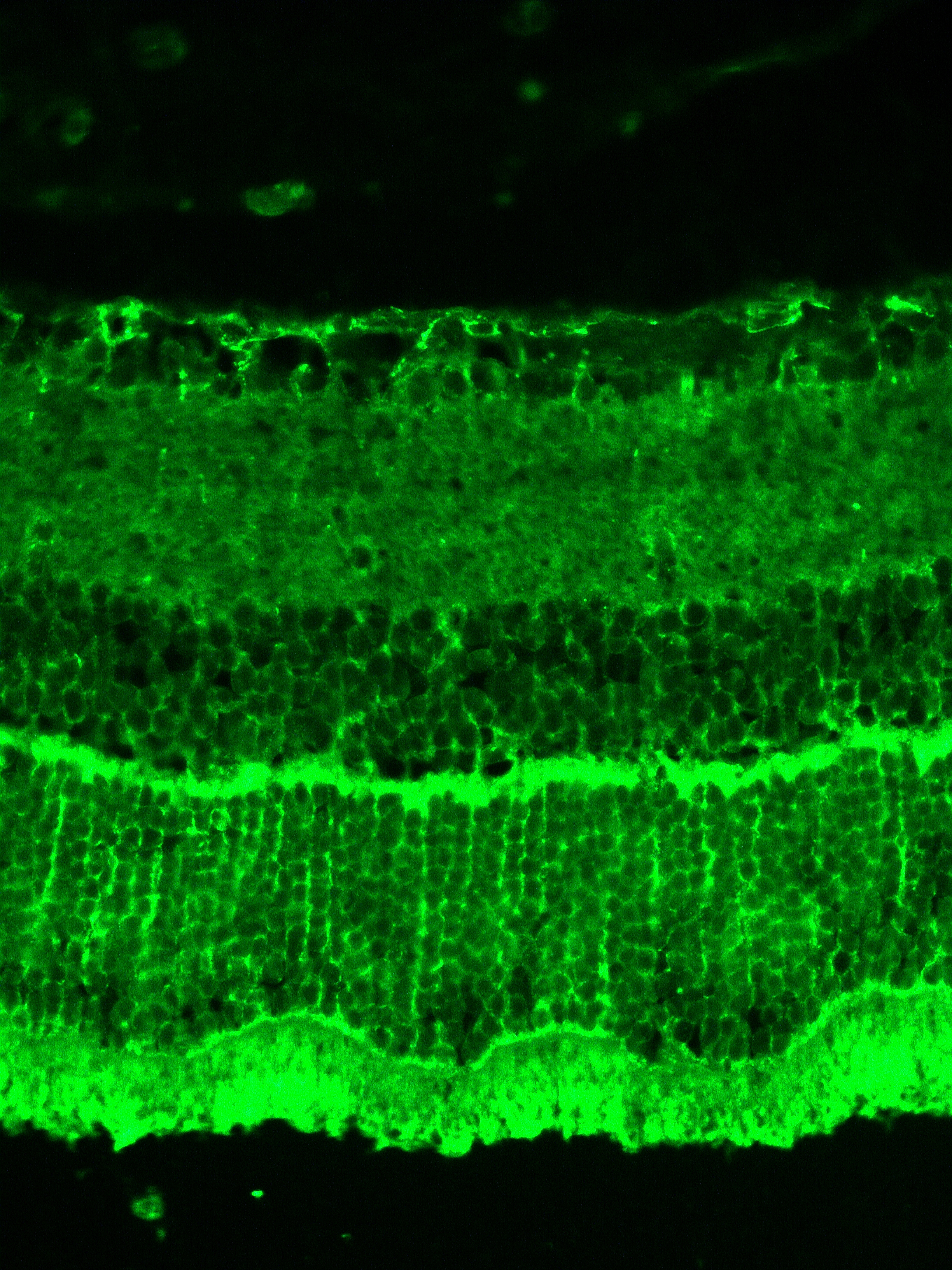

Supplement: S12 Fig — (JPG) [file pone.0156495.s012.jpg]

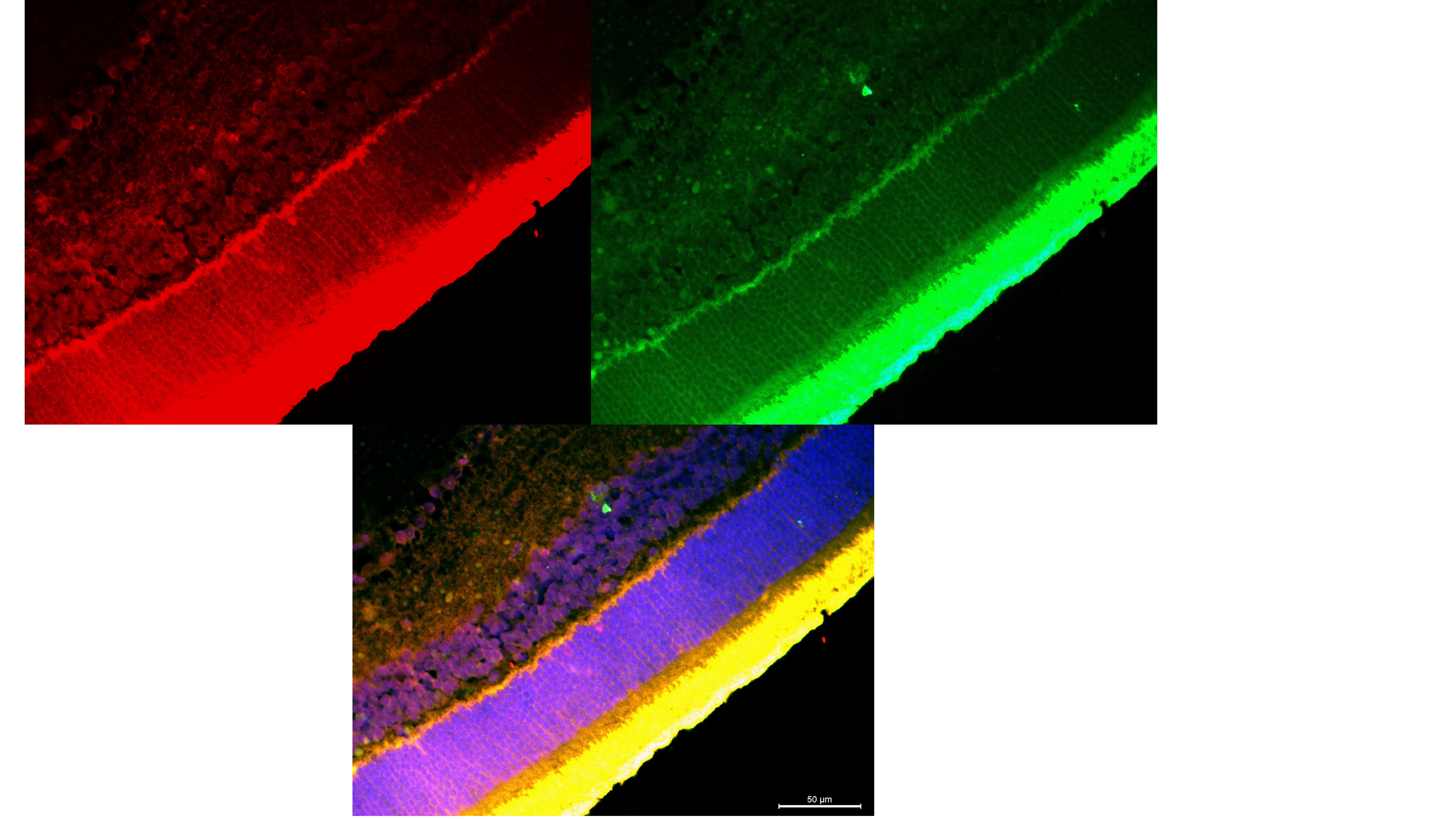

Supplement: S13 Fig — (JPG) [file pone.0156495.s013.jpg]

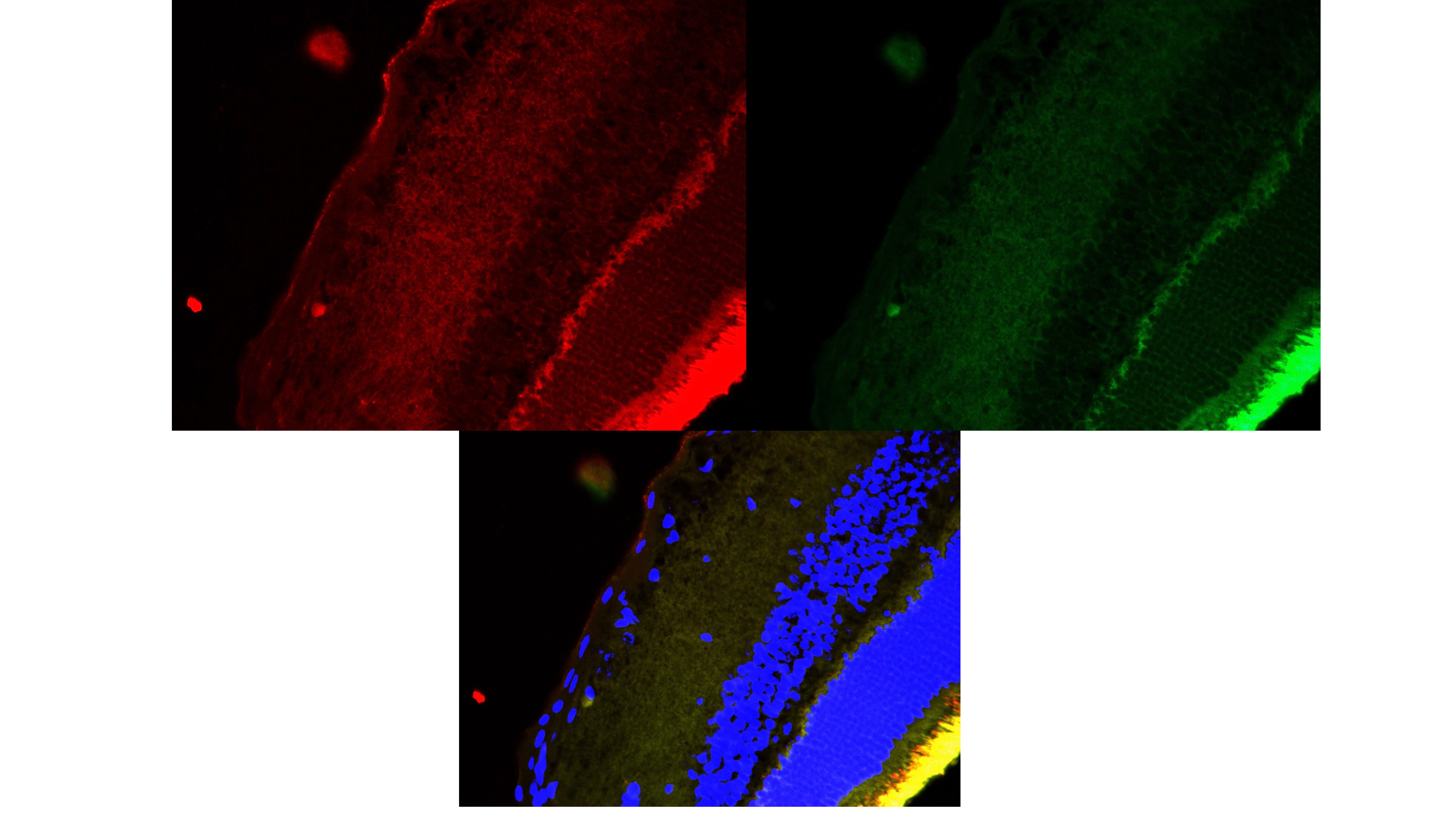

Supplement: S14 Fig — (JPG) [file pone.0156495.s014.jpg]
